# Supplementary figures and images for: Respiratory syncytial virus ribonucleoproteins hijack microtubule Rab11 dependent transport for intracellular trafficking
Source: PLoS Pathog. 2022 Jul 7;18(7):e1010619. doi: 10.1371/journal.ppat.1010619 (PMC9262236; doi:10.1371/journal.ppat.1010619)

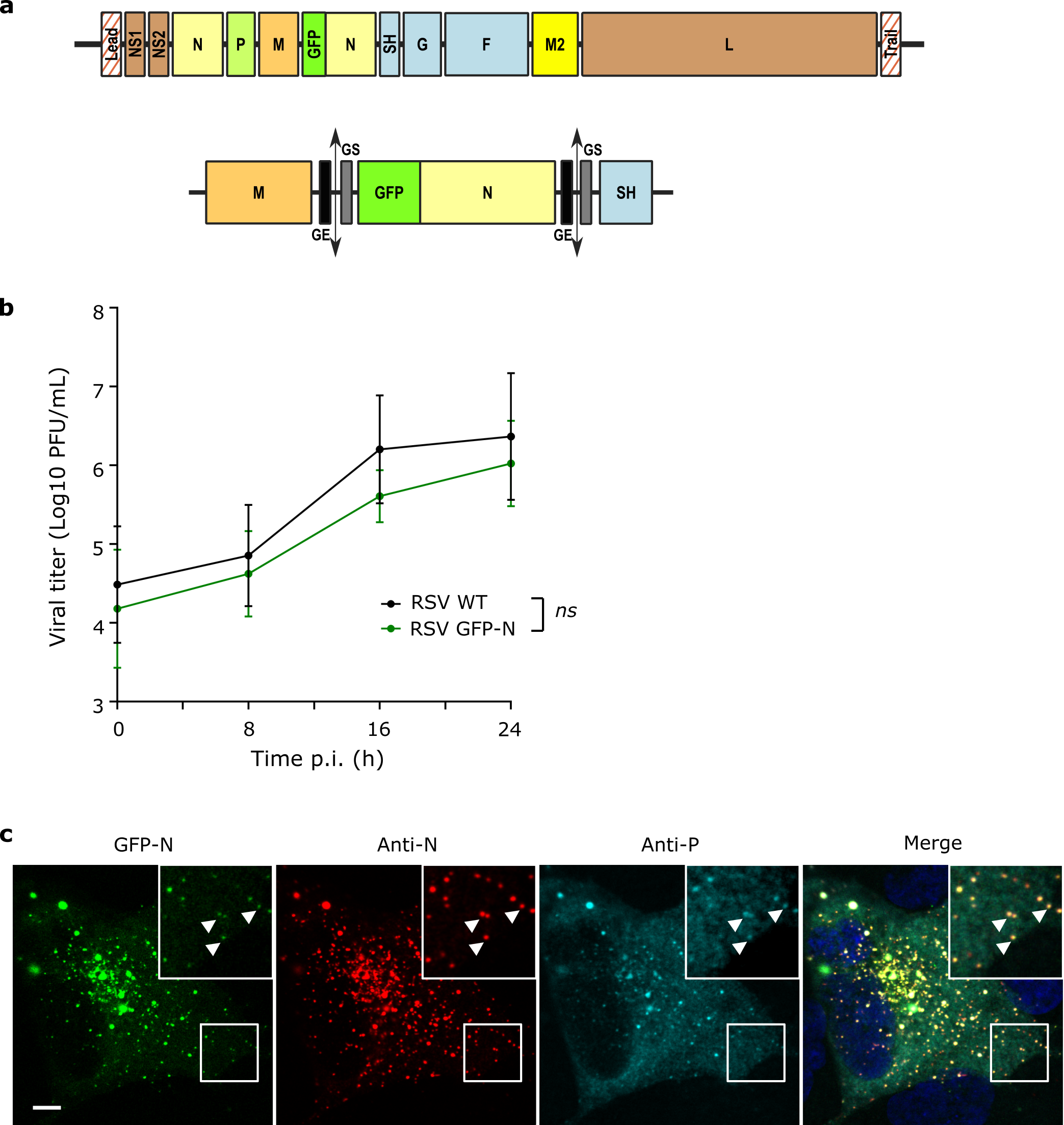

Supplement: S1 Fig — a) Schematic representation of RSV-GFP-N infectious clone (not to scale). Protein-encoding frames are shown as colored boxes, leader and trailer regions as hatched boxes. Intergenic regions are shown as black horizontal lines. The GFP-N coding sequence was inserted between M and SH genes together with an upstream gene end (GE) and a downstream gene start (GS) signal as described in Methods. b) Growth properties of RSV-GFP-N. HEp-2 cells were infected with the RSV-GFP-N or RSV WT at a MOI of 1 at 37°C and viruses were harvested at the indicated times p.i. and titrated by plaque assays on HEp-2 cells. Results are the mean ± s.d. for three independent experiments. Titers of RSV-GFP-N and RSV at the different time points are not significantly different using two-way ANOVA (ns for RSV versus RSV GFP-N; ns for interaction) followed by Sidak’s multiple comparison test. c) Colocalization of GFP-N with wild type N and P proteins in RSV-GFP-N infected cells. HEp-2 cells were infected with RSV-GFP-N. At 24h p.i. cells were stained with antibodies against N (red) and P (cyan) and Hoechst 33342 (merge). The GFP-N protein is visualized through its spontaneous green fluorescence. RNPs are indicated with white arrow heads. Representative images are shown. Images were taken under a Leica SP8 confocal microscope. Images stacks (3 z-steps) were processed as maximum projections and visualized after gaussian filter fixed at 0.5. Scale bar 5 μm. (TIF) [file ppat.1010619.s001.tif]

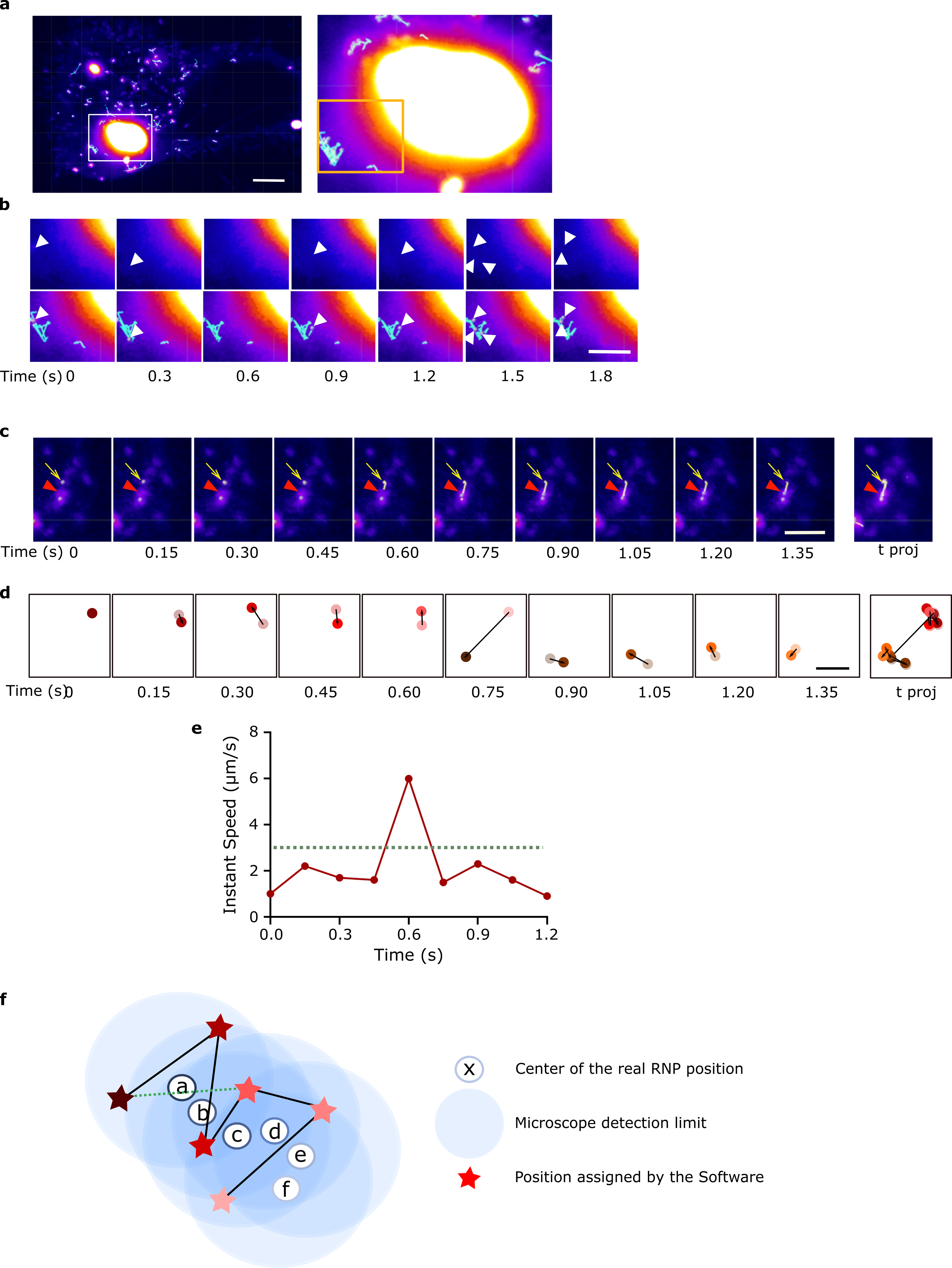

Supplement: S2 Fig — a, b) Illustration of false tracking near the IBs. a) Time projection of 14 consecutives frames over 1.95 s showing spot detections (grey spheres) and tracking (cyan dragon tails) by Imaris software on RSV-GFP-N infected HEp-2 as described in methods section. Artifactual tracks are visible near the large IB. Scale bar 5μm. A zoom of the region is shown on the right. b) Consecutives images of the area framed in orange (for clarity one out of two is removed). The top panel shows the original images, the lower panel shows the images with spots and track marks. Detected spots used to generate the tracks are pointed with white arrows on both panels. Note that no spots are visible on the original images. Tracks detected in the framed region on panel a) will be removed from the analysis. Scale bar 2μm c, d, e) Illustration of false tracking due to wrong link between 2 slow moving objects. c) Consecutive images showing spot detections (grey spheres) and tracking (yellow dragon tail) by Imaris software on RSV-GFP-N infected HEp-2 as described in methods section. In this example, the first detected particle is pointed by the yellow arrow and is animated with slow undirected motion. At time 0.75 s a new spot is detected (red arrow) and is associated with the track of the first one. This second spots exhibits also slow motion. Yellow and red arrows are pointing the same position on every images. Scale Bar 2μm. The spot is supposed to have covered approximately 0.8μm in 0.15 s resulting in an instant velocity of 5.3μm/s between these positions. This track will be filtered because all other instant velocities are below 2.6 μm/s (50% of the track maximum instant velocity). d, e) Schematic of the false tracking resulting from a wrong link between 2 slow moving spots. In each frame, previous position is indicated in fainted color and displacement is indicated by a black arrow. Scale bar 0.5μm. At time 0.75s the previous position of the first spot is linked to another particle positi [file ppat.1010619.s002.tif]

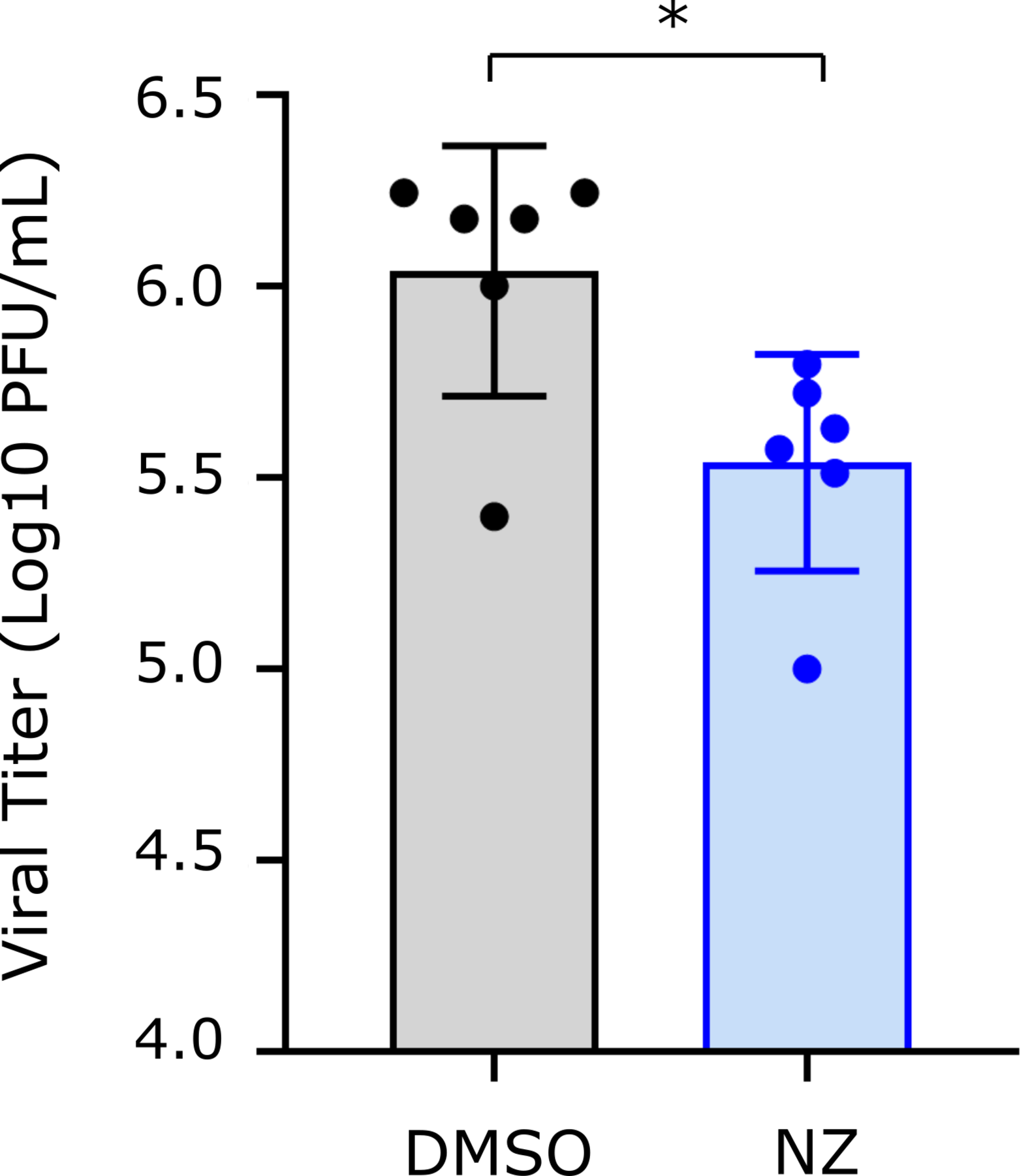

Supplement: S3 Fig — A549 cells were infected with RSV at high MOI and Nocodazole 2μM (NZ) or DMSO was added 2 h post infection. At 24h viral titer of each sample was determined by plaque assay. Mean ± s.d. from 2 experiments in triplicate are shown. * p <0.05 using Kolmogorov-Smirnov comparison test. (TIF) [file ppat.1010619.s003.tif]

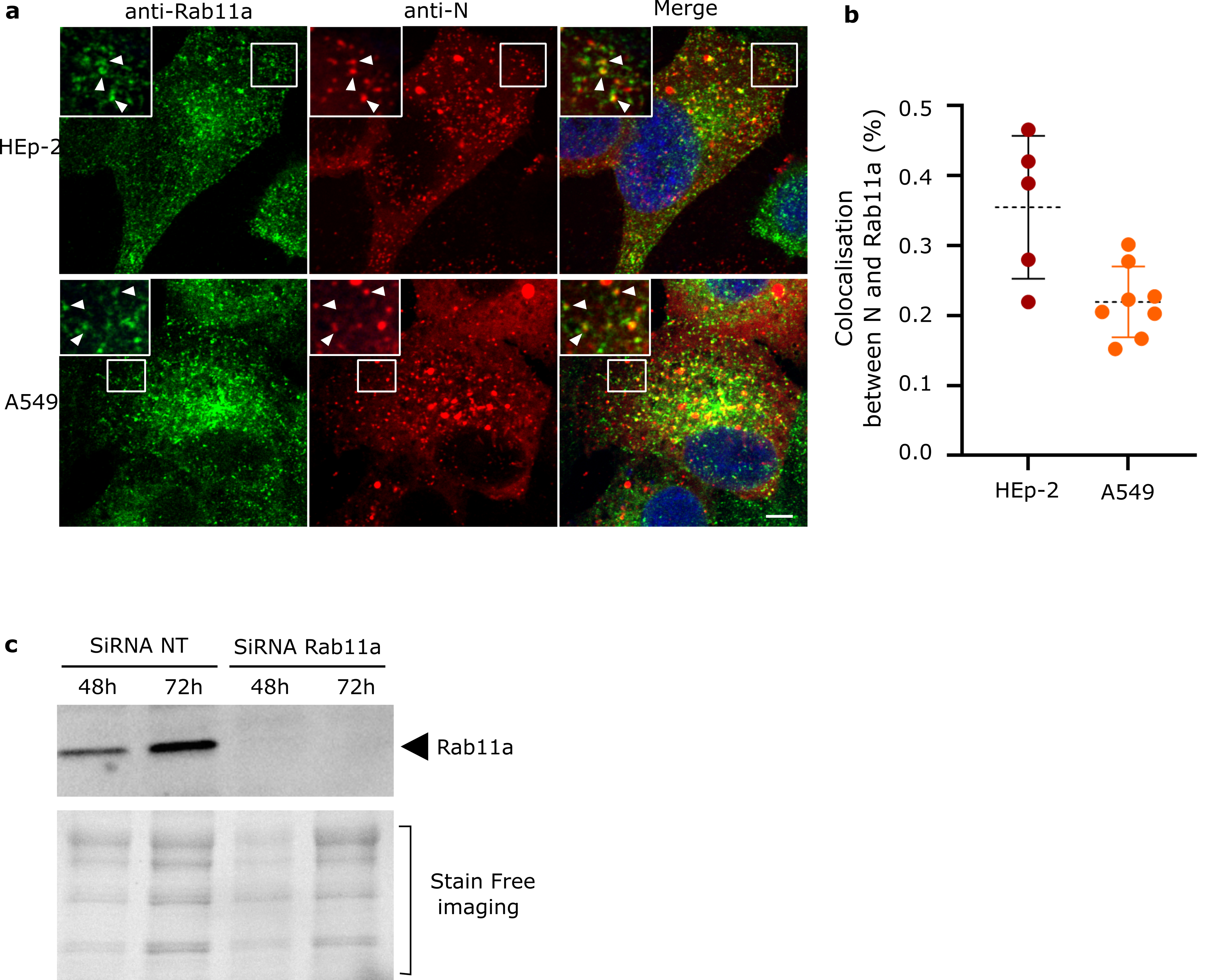

Supplement: S4 Fig — a) HEp-2 and A549 cells infected with RSV for 24 h. Rab11a (green) and RNPs (red) were detected by immunostaining. Representative images from one experiment are shown. Images stacks (3 z-steps) were processed as maximum projections and visualized after gaussian filter fixed at 0.5. Scale bar 5 μm. The boxed areas enclose Rab11 and N spots pointed by white arrows (zoom). b) Percentage of N spots colocalizing with Rab11a positive spots calculated using Icy Software. Results from individual cells from one experiment are plotted with mean and s.d. c) Extinction of Rab11a expression in SiRNA transfected cells. A549 cells were treated by siRNA Rab11a or non-targeting siRNA (control) for 48 h and 72h. Cell lysates were subjected to SDS-PAGE and probed by antibodies directed against Rab11a. The visualization of all proteins was realized by Stain Free revelation. (TIF) [file ppat.1010619.s004.tif]

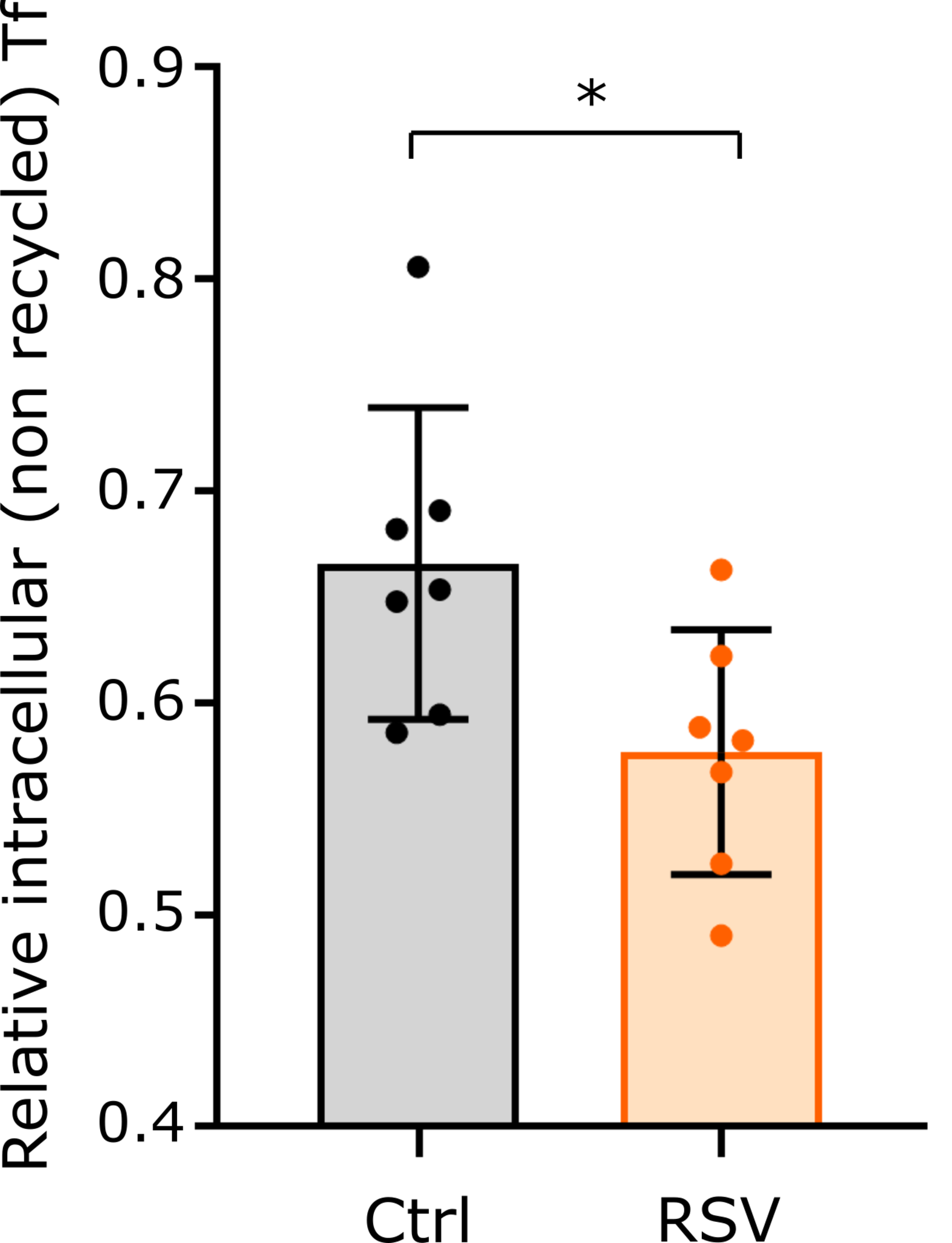

Supplement: S5 Fig — HEp-2 cells infected with RSV GFP or mock infected for 24 hours were incubated with Alexa-647-Tf for 10min then washed and incubated in fresh medium for 0 to 20 min. Cells were fixed and observed under confocal microscopy. Total amount of Alexa-647-Tf per cell was quantified. Mean ± s.d. of relative amount of Alexa 647 Tf after 20 min of chase is shown. * p< 0.05 using t test with Welch’s correction. Data are from 7 cells from one representative experiment out of 2. (TIF) [file ppat.1010619.s005.tif]
