# Supplementary material for: Respiratory syncytial virus ribonucleoproteins hijack microtubule Rab11 dependent transport for intracellular trafficking
Source: PLoS Pathog. 2022 Jul 7;18(7):e1010619. doi: 10.1371/journal.ppat.1010619 (PMC9262236; doi:10.1371/journal.ppat.1010619)
Supplement: Attachment — Submitted filename: Cosentino PPATHOGENS-D-22-00304_reviewer suggestions 032122.pdf [file ppat.1010619.s017.pdf]

PLOS Pathogens

Respiratory syncytial virus ribonucleoproteins hijack microtubule Rab11 dependent transport for intracellular trafficking

--Manuscript Draft--

**Manuscript Number:**	PPATHOGENS-D-22-00304
**Full Title:**	Respiratory syncytial virus ribonucleoproteins hijack microtubule Rab11 dependent transport for intracellular trafficking
**Short Title:**	RSV ribonucleoproteins export relies on microtubules and recycling endosome pathway
**Article Type:**	Research Article
**Section/Category:**	Virology
**Keywords:**	respiratory syncytial virus; mononegavirales; live imaging; ribonucleoprotein trafficking; ribonucleoprotein export; intracellular trafficking; automatic tracking, recycling endosome
**Abstract:**	The respiratory syncytial virus (RSV) is the primary cause of severe respiratory infection in infants worldwide. Replication of RSV genomic RNA occurs in cytoplasmic inclusions generating viral ribonucleoprotein complexes (vRNPs). vRNPs then reach assembly and budding sites at the plasma membrane. However, mechanisms ensuring vRNPs transportation are unknown. We generated a recombinant RSV harboring fluorescent RNPs allowing us to visualize moving vRNPs in living infected cells and developed an automated imaging pipeline to characterize the movements of vRNPs at a high throughput. Automatic tracking of vRNPs revealed that around 10% of the RNPs exhibit fast and directed motion compatible with transport along the microtubules. Visualization of vRNPs moving along labeled microtubules and restriction of their movements by microtubule depolymerization further support microtubules involvement in vRNPs trafficking. Approximately 30% of vRNPs colocalize with Rab11a protein, a marker of the endosome recycling (ER) pathway and we observed vRNPs and Rab11-labeled vesicles moving together. Transient inhibition of Rab11a expression significantly reduces vRNPs movements demonstrating Rab11 involvement in RNPs trafficking. Finally, Rab11a is specifically immunoprecipitated with vRNPs in infected cells suggesting an interaction between Rab11 and the vRNPs. Altogether, our results strongly suggest that RSV RNPs move on microtubules by hijacking the ER pathway.
**Additional Information:**	
**Question**	**Response**
**Financial Disclosure**<br><br>Enter a financial disclosure statement that describes the sources of funding for the work included in this submission. Review the submission guidelines for detailed requirements. View published research articles from *PLOS Pathogens* for specific examples.<br><br>This statement is required for submission and **will appear in the published article** if the submission is accepted. Please make sure it is accurate.	MARW was supported by ATIP-AVENIR INSERM program (2018)(https://www.inserm.fr/nous-connaitre/programme-atip-avenir/), and the Fondation Del Duca - Institut de France (https://www.fondation-del-duca.fr/). The funders had no role in study design, data collection and analysis, decision to publish, or preparation of the manuscript.

Powered by Editorial Manager® and ProduXion Manager® from Aries Systems Corporation

**Unfunded studies**<br>Enter: *The author(s) received no specific funding for this work.*<br><br>**Funded studies**<br><br>Enter a statement with the following details:<br>• Initials of the authors who received each award<br>• Grant numbers awarded to each author<br>• The full name of each funder<br>• URL of each funder website<br>• Did the sponsors or funders play any role in the study design, data collection and analysis, decision to publish, or preparation of the manuscript?<br>• **NO** - Include this sentence at the end of your statement: *The funders had no role in study design, data collection and analysis, decision to publish, or preparation of the manuscript.*<br>• **YES** - Specify the role(s) played.<br><br>* typeset	
**Competing Interests**<br><br>Use the instructions below to enter a competing interest statement for this submission. On behalf of all authors, disclose any competing interests that could be perceived to bias this work—acknowledging all financial support and any other relevant financial or non-financial competing interests.<br><br>This statement **will appear in the published article** if the submission is accepted. Please make sure it is accurate. View published research articles from *PLOS Pathogens* for specific examples.	The authors have declared that no competing interests exist.

**NO authors have competing interests**<br><br>Enter: *The authors have declared that no competing interests exist.*<br><br>**Authors with competing interests**<br><br>Enter competing interest details beginning with this statement:<br><br>*I have read the journal's policy and the authors of this manuscript have the following competing interests: [insert competing interests here]*<br><br>* typeset<br><br>This statement is required for submission and **will appear in the published article** if the submission is accepted. Please make sure it is accurate and that any funding sources listed in your Funding Information later in the submission form are also declared in your Financial Disclosure statement.	
**Data Availability**<br><br>Authors are required to make all data underlying the findings described fully available, without restriction, and from the time of publication. PLOS allows rare exceptions to address legal and ethical concerns. See the PLOS Data Policy and FAQ for detailed information.<br><br>A Data Availability Statement describing where the data can be found is required at submission. Your answers to this question constitute the Data Availability Statement and **will be published in the article**, if accepted.<br><br>**Important:** Stating 'data available on request from the author' is not sufficient. If your data are only available upon request, select 'No' for the first question and explain your exceptional situation in the text box.	Yes - all data are fully available without restriction

Do the authors confirm that all data underlying the findings described in their manuscript are fully available without restriction?	
**Describe where the data may be found in full sentences. If you are copying our sample text, replace any instances of XXX with the appropriate details.**<br>• If the data are **held or will be held in a public repository**, include URLs, accession numbers or DOIs. If this information will only be available after acceptance, indicate this by ticking the box below. For example: *All XXX files are available from the XXX database (accession number(s) XXX, XXX.*).<br>• For research data submitted to Dryad, enter the following: *The data is available from Dryad repository DOI: XXX*.<br>• If the data are all contained **within the manuscript and/or Supporting Information files**, enter the following: *All relevant data are within the manuscript and its Supporting Information files.*<br>• If neither of these applies but you are able to provide **details of access elsewhere**, with or without limitations, please do so. For example:<br><br>*Data cannot be shared publicly because of [XXX]. Data are available from the XXX Institutional Data Access / Ethics Committee (contact via XXX) for researchers who meet the criteria for access to confidential data.*<br><br>*The data underlying the results presented in the study are available from (include the name of the third party and contact information or URL).*<br>• This text is appropriate if the data are owned by a third party and authors do not have permission to share the data.<br><br>* typeset	All relevant data are within the manuscript and its Supporting Information files.<br>The nucleotide sequence of RSV-GFP-N was deposited in the Genbank nucleotide database with accession code OM326756<br>The dedicated PYTHON Script is available at GitHub (https://github.com/mawelti/RSV-RNP-TrackAnalysis)
Additional data availability information:	

Click here to access/download;Manuscript;Cosentino_et_al-manuscript - plos.docx

Respiratory syncytial virus ribonucleoproteins hijack microtubule Rab11 dependent transport for intracellular trafficking

Gina Cosentino[1], Katherine Marougka[1], Aurore Desquesnes[1], Nicolas Welti[1], Delphine Sitterlin[1], Elyanne Gault[2], Marie-Anne Rameix-Welti[2]

[1] Université Paris-Saclay, Université de Versailles St. Quentin, UMR 1173 (2I), INSERM, Versailles, France.

[2] Université Paris-Saclay, Université de Versailles St. Quentin; UMR 1173 (2I), INSERM ; Assistance Publique des Hôpitaux de Paris, Hôpital Ambroise Paré, Laboratoire de Microbiologie, DMU15 ; Versailles, France.

Short title: RSV ribonucleoproteins export relies on microtubules and recycling endosome pathway

Abstract

The respiratory syncytial virus (RSV) is the primary cause of severe respiratory infection in infants worldwide. Replication of RSV genomic RNA occurs in cytoplasmic inclusions generating viral ribonucleoprotein complexes (vRNPs). vRNPs then reach assembly and budding sites at the plasma membrane. However, mechanisms ensuring vRNPs transportation are unknown. We generated a recombinant RSV harboring fluorescent RNPs allowing us to visualize moving vRNPs in living infected cells and developed an automated imaging pipeline to characterize the movements of vRNPs at a high throughput. Automatic tracking of vRNPs revealed that around 10% of the RNPs exhibit fast and directed motion compatible with transport along the microtubules. Visualization of vRNPs moving along labeled microtubules and restriction of their movements by microtubule depolymerization further support microtubules involvement in vRNPs trafficking. Approximately 30% of vRNPs colocalize with Rab11a protein, a marker of the endosome recycling (ER) pathway and we observed vRNPs and Rab11-labeled vesicles moving together. Transient inhibition of Rab11a expression significantly reduces vRNPs movements demonstrating Rab11 involvement in RNPs trafficking. Finally, Rab11a is specifically immunoprecipitated with vRNPs in infected cells suggesting an interaction between Rab11 and the vRNPs. Altogether, our results strongly suggest that RSV RNPs move on microtubules by hijacking the ER pathway.

Author summary

Respiratory syncytial virus is the leading cause of severe lower respiratory infection in children worldwide and is increasingly recognized as a major respiratory pathogen in the elderly and frail. Yet, no curative treatment or vaccine is currently marketed. The late stages of RSV multiplication remain poorly understood despite their potential targets for the development of antiviral strategies. In the infected cell, the viral genome is encapsidated and associated to the viral polymerase, to form the viral ribonucleoprotein (vRNP). The vRNPs are produced and assembled in cytoplasmic viral factories. The process ensuring their transport to the budding sites, at the plasma membrane, awaits to be precisely defined. Here we explored these mechanisms by tracking moving vRNPs in living infected cells. We developed an automated imaging pipeline allowing us to characterize the movements of vRNPs, at an unprecedented throughput. We then exploited the potential of our method to monitor the behaviour of the vRNPs during the infection. Using this approach, we document substantial trafficking of RSV along the microtubule network and demonstrate that RSV hijacks the recycling endosome pathway to promote the mobility of its vRNPs. Altogether, this work provides a cutting-edge approach allowing for

live visualization of RSV RNP trafficking and critical data toward the understanding of RSV RNPs movements.

Introduction

Respiratory syncytial virus (RSV) is the leading cause of severe lower respiratory tract infection in children worldwide. RSV infections are responsive for around 120 000 child death per year mostly in developing countries and are the main cause of child's hospitalization in western countries (1,2). Periodic reinfections occur throughout life. Considered as benign in healthy children and adults, RSV infections are increasingly associated with significant morbidity and mortality in elderly and immunocompromised people with much the same disease burden as for influenza (3–5). Despite the high burden of RSV infection, there are still no vaccine nor curative treatment available. Search for antiviral drugs is active, most of the candidates in development targeting entry steps and viral RNA synthesis (6).

RSV belongs to the *Mononegavirales* order. Its 15 kb negative single stranded genomic RNA encode 11 proteins. RSV is an enveloped virus exhibiting two major surface glycoproteins G and F. The matrix protein M coats the inner side of the viral membrane and surrounds the viral genomic material (7). The genomic RNA is tightly encapsidated by the nucleoprotein N and forms an helicoidal ribonucleocapsid. N is further bound to the polymerase (L) and its viral cofactors, the phosphoprotein P and M2-1, to form the viral ribonucleoprotein (vRNP) (8–10). RNPs are the functional units driving viral RNA synthesis. Using the ribonucleocapsid as a template, L and P proteins ensure both the viral transcription and replication processes, while M2-1 is selectively required for the transcription process. Viral RNA synthesis occurs within cytoplasmic inclusions, referred to as inclusion bodies (IBs), which can be regarded as viral factories (11). The newly synthetized RNPs then reach the plasma membrane, where RSV presumably buds, forming elongated membrane filaments (12–14). The matrix protein M is thought to drive virion assembly by bridging RNPs with the plasma membrane through its interactions with the RNPs and the cytoplasmic tail of the viral F glycoprotein (14–19). The exact location of final virus assembly is debated since recent analysis of live G trafficking showed that RNP assemble with membrane glycoprotein prior insertion to plasma membrane (20). In any case, trafficking of RNPs to the cell surface requires active transport mechanisms, since diffusion of large objects in the cytoplasm is restricted by molecular crowding by organelles, the cytoskeleton and high protein concentrations (21). This active transport might involve hijacking of the actin networks (22,23) or of the recycling endosome (RE) pathway. RE is involved in delivering endocytosed material as well as cargos from the trans Golgi network to the plasma membrane (24). Interestingly RE pathway has been implicated in RNP export of numerous viruses such as the influenza, Sendai, measles and mumps viruses (25–28). The RE pathway is regulated by the small GTPase Rab11 which is present in three isoforms (Rab11a, Rab11b and Rab25) in human cells. The GTP bound active form of Rab11 binds to target vesicle' membrane thanks to its GPI anker and recruits specific factors ensuring transportation,

docking or fusion of the vesicle to its cognate membrane (29). Rab11 vesicle can traffic along both microtubules and actin networks by engaging various molecular motors by means of molecular adaptors called Rab11 Family Interacting Proteins (Rab11-FIPs).

Here we engineered recombinant RSV expressing a fluorescent N to visualize the moving RNPs in living infected cells. An automatic quantitative analysis of RNPs trajectories reveals rapid and directional motions that were abolished by nocodazole treatment, consistent with transportation along the microtubule network. We observed a colocalization of around one third of the RNPs with Rab11a and RNPs moving together with Rab11a in infected cells. Inhibition of Rab11a expression impairs RNPs movements. Interestingly RSV infection affects transferrin recycling, dependent of Rab11 pathway. Moreover, interaction between Rab11a and viral RNPs was evidenced by co-immunoprecipitation experiments. All of these data confirm that RSV is high jacking the Rab11-RE pathway to ensure RNPs export.

Results

Development of recombinant RSV to monitor intracellular transport of RNPs.

To investigate RNPs dynamics in living cells, the RSV N protein was fused to a fluorescent tag to generate fluorescent RNPs. We previously demonstrated that a N-ter fusion of the N doesn't affect the transcription-replication activities if co-expressed with a wild type N (11). We thus engineered and rescued a recombinant RSV expressing both the wild type N and a GFP-N by inserting the GFP-N coding sequence flanked with transcription start and stop signals sequences between M and SH genes in our reverse genetic vector (Fig S1a) (30). To ascertain whether the GFP-N would interfere with viral growth, we compared single cycle growth kinetics of wild type and N-GFP expressing RSV (hereinafter referred to as RSV-GFP-N) (Fig S1b). The RSV-GFP-N growth was similar to the wild type RSV and reached titers around 10^6 PFU/mL in 24h, showing that the GFP-N addition did not significantly impair viral multiplication. We then investigated GFP-N protein localization in respect to the wild type N protein and known viral structures such as IBs and viral filaments. HEp-2 cells were infected by RSV-GFP-N for 24h and intracellular localization of RSV N, P and F proteins were determined by confocal imaging after immunostaining of each protein. The GFP signals were recorded in parallel. As previously described, IBs, cytoplasmic dots and viral filaments were observed by immunostaining of N and P, and F staining only revealed viral filaments (Fig 1) (31). The GFP signals perfectly colocalize with N and P staining in IBs, viral filaments and small puncta in the cytoplasm. Of particular interest, we noted a perfect colocalization of GFP signals with both N and P staining in cytoplasmic dots which could be considered as RNPs (Fig 1 and S1c). Moreover, we observed colocalization of the GFP signals with F staining in viral filaments (Fig 1c, panel F). These data strongly suggest that GFP-N is incorporated together with the wild type N in the vRNPs. Altogether, the RSV-GFP-N was considered suitable as a novel tool marker to assess vRNPs trafficking across the cytoplasm during infection.

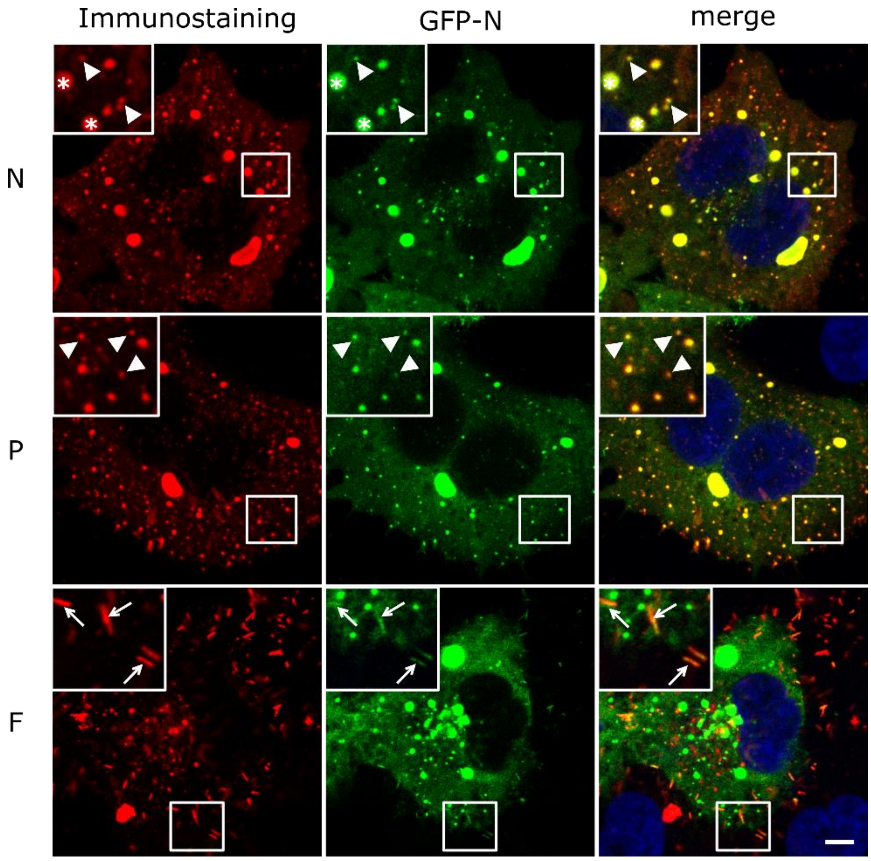

Figure 1: Localization of GFP-N and wild type N, P and F proteins in RSV-GFP-N infected cells. HEp-2 cells were infected with RSV-GFP-N. At 24h p.i. cells were stained with antibodies against N, P or F (red) and Hoechst 33342 (merge). The GFP-N protein was visualized through its spontaneous green fluorescence. IBs are indicated with a star, RNPs are indicated with white arrow heads and viral filaments are indicated with white arrows. Representative images from 2 independent experiments are shown. Images stacks (3 z-steps) were processed as maximum projections and visualized after gaussian filter fixed at 0.5. Scale bar 5 µm.

Dynamics of RNPs in RSV infected cells

To assess RNPs dynamics in living cells, we analyzed HEp-2 or A549 cells infected with RSV-GFP-N for 18-20h by video-microscopy. High-rate time-lapse images were acquired under a confocal spinning disk microscope (Supplementary Movie 1). Numerous small GFP positive dots are visible. Most dots exhibited slow motion movements with rapid and sudden changes of direction when some exhibited fast directed trajectories (white arrow head Fig 2a). Strikingly fast-moving particles appeared stretched (Fig 2a). Automatic single particle tracking of the GFP positive dots was undertaken using Imaris software (Supplementary Movie 2) and resulted in hundreds of tracks per cells (266 to 2342, mean 993 from 23 cells in 4 independent experiments). Tracks located nearby IBs were removed from analysis as slight changes of fluorescence within IBs were prone to induce false-positive signals (Fig S2a). We also filtered tracks, in which particles instant speeds always remained below 50% of their maximal instant speed along the entire track recorded to remove artifactual links between the tracks of two slow moving objects (See Fig S2b and legend).

We then quantified several parameters characterizing intracellular movement of vRNPs. Displacement is characterized by 1) the *track length* which corresponds to the sum of lengths of displacement between two time points and 2) the *track displacement*, which corresponds to the minimum distance between the first and the last position of the particle (Fig 2b). *Track velocity* is the ratio between track displacement and track duration. Track displacement and track velocity are thus expected to be high for particles exhibiting long range-oriented motion. *Smoothed instant speed* is the ratio between the minimum distance between the first and the fourth of 4 consecutive positions and the time interval between these positions (see Fig S2f and corresponding legend). Focusing on instant velocities of single fast-moving particles, we observed that rapid motions occur intermittently (Fig 2c). Thus, we also calculated the *track maximum speed* corresponding to the maximum smooth instant speed of the track. Cumulative distribution of track displacement for 6 cells in one experiment is shown in Figure 2e. Around 10% of the tracked particles exhibit a displacement over 1.8 µm when most of them exhibit small displacement (median ranging from 0.29 to 0.42µm). Likewise, cumulative analysis of particles maximal speeds shows that around 10% of the RNPs exhibit maximum velocities above 1.7µm/s (Fig 2d). The median value of each parameter reflects the overall characteristics of the particle's movements in an individual cell and provides a single value that can be used for further comparing treated groups. Median of track maximal speeds, track velocities and track displacements are not significantly different in 4 independent experiments on two different cell types (HEp-2 or A549). This points to a potential generalizability of vRNP's behavior during RSV infection in this model and indicates that these parameters are suitable for further group comparisons (Fig 2f, 2g, 2h).

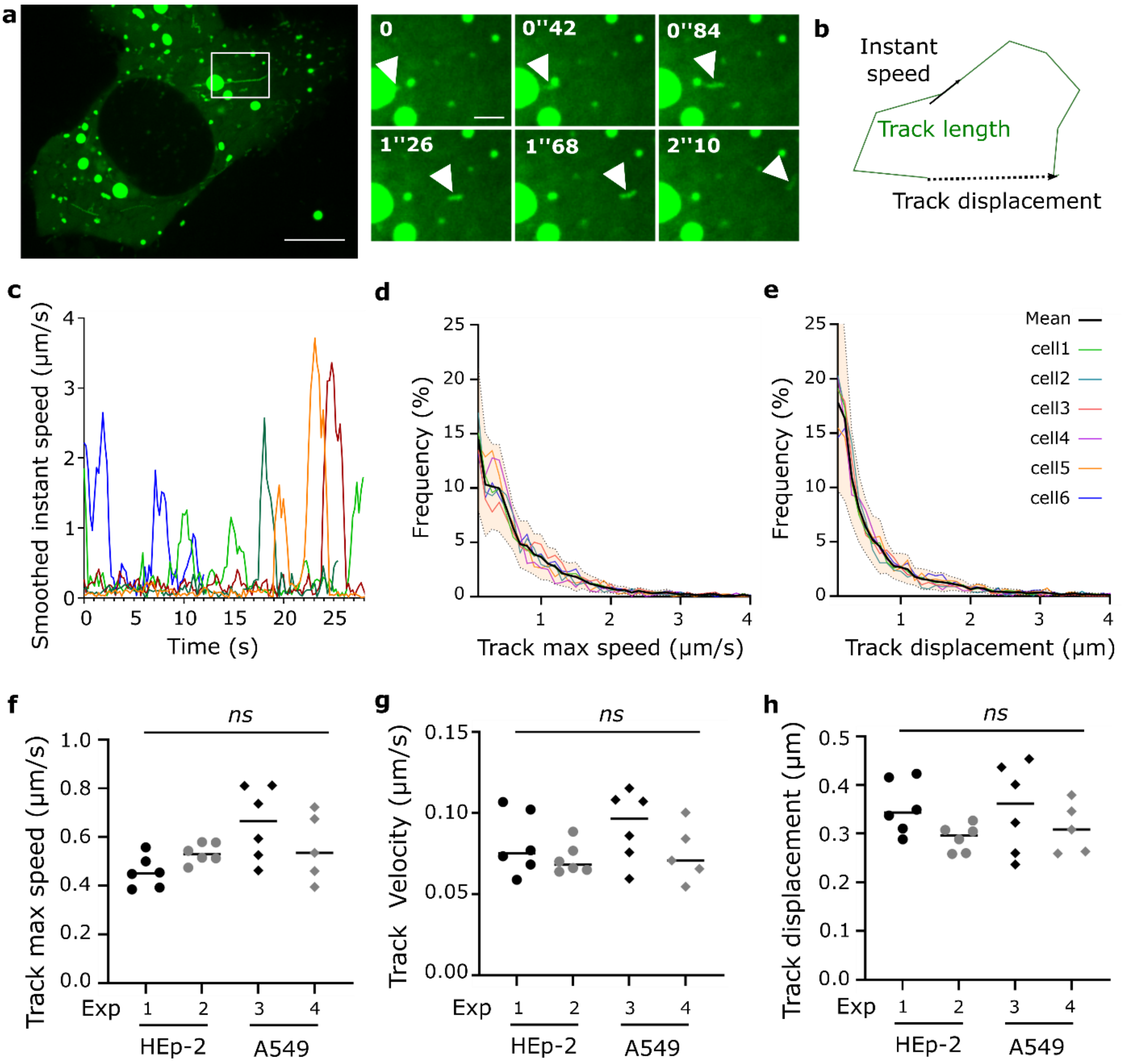

Figure 2. Characterization of RNPs intracellular motions in RSV-GFP-N infected cells. Live images of HEp-2 or A549 cells infected with RSV-GFP-N for 18 to 20h are analyzed using Imaris software as described in methods section. **a)** Dynamic behavior of RSV RNPs in HEp-2 cells. Images stacks (3 z-steps) were processed as maximum projections and visualized after gaussian filter fixed at 0.5, the large view is a time projection of 12 consecutive frames (0.21 s between frames). Scale bar 10 µm. The zoomed time series show a fast-moving RNP pointed by white arrow heads, scale bar 2 µm. Representative images from 12 movies from 2 independent experiments are shown. **b)** Schematic illustration of the motion characterization parameters (instant speed, track length, track displacement). **c)** Smoothed instant speeds of fast-moving particles plotted versus time from the first detection (each color represents one particle, representative examples). **d, e)** Cumulative distribution of track max speed and track displacement from 6 individual HEp-2 cells from one experiment. **f, g and h)** Each point is the median of track maximum speed, track velocity and track displacement from an individual cells. *ns*: No statistical difference between 4 independent experiments on HEp-2 and A549 cells using Brown-Forsythe and Welch's ANOVA test.

RSV RNPs rapid movements are dependent on MT network

Directional, rapid and discontinuous movements of the vRNPs are suggestive of microtubule (MT)-dependent transport (32). We investigated vRNPs localization relative to MT in HEp-2 and A549 cells infected with wild type RSV for 20h. N and MT were immunostained and cells were analysed on a super resolution confocal microscope (Airyscan, Leica). High-resolution images of infected cells revealed that MT were decorated with multiple vRNPs (Fig 3). To visualize the movements of vRNPs relative to MT, RSV-GFP-N infected A549 cells were treated with docetaxel –AF 647, a fluorescent-linked drug binding MT polymers. This enabled MT live staining and dual colored time-lapse imaging acquisition. Fluorescent vRNPs moving rapidly along microtubules structures were clearly visible demonstrating RNPs transport along MT network (Supplementary Movie 3 and Fig 4a). Acquisition in two color channels prevented any quantitative analysis of vRNPs trajectories. To further dissect the involvement of MT network in RNPs movements, we analyzed the effects of MT depolymerisation on RNPs movements. Living RSV-GFP-N infected cells were imaged before and after a 10 min treatment with nocodazole (20µM). Importantly, our protocol allowed to image the same cells before and after drug-treatment. Remarkably, the nocodazole treatment abolished almost all the rapid and directed movements (Supplementary Movies 4). In contrast, cells treated with Cytochalasin D, a drug impairing actin polymerization, still displayed fast moving vRNPs (Supplementary Movies 5). Finally, we applied our pipeline of GFP particles tracking on mock or nocodazole treated cells, and observed a significant decrease of track displacement, track velocity and track maximum speed (Fig 4d, 4e, 4f, Supplementary Movies 6 and 7). A centered projection of the tracks of one cell during 60s is shown in figure 4 to illustrate the strong reduction of track displacement in nocodazole treated cells. Altogether, these data revealed that fast-directed movement of RNPs rely on MT network. Consistent with the role of MT in RNPs transportation, nocodazole treatment of RSV infected cells after entry steps significantly decrease progeny virus production as previously reported (Fig S3) (33).

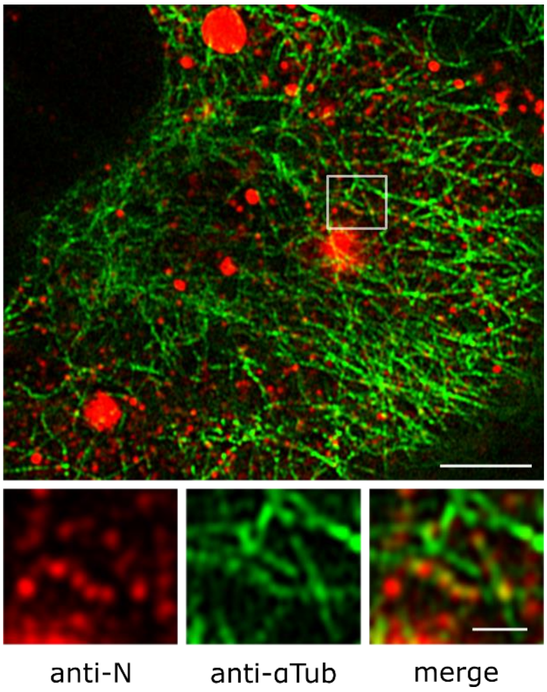

Figure 3: RNPs are decorating the MT network. A549 cells were infected with RSV for 20h. N (in red) and α-Tubulin (in green) are revealed by immunostaining. High-resolution images were generated using a confocal microscope with Airyscan detector. Images were visualized after gaussian filter fixed at 0.5. A representative image of a whole cell out of 5 cells in 3 experiments is shown. RNPs decorating the MT network are shown on the zoom area. Scale bars 5µm and 1 µm.

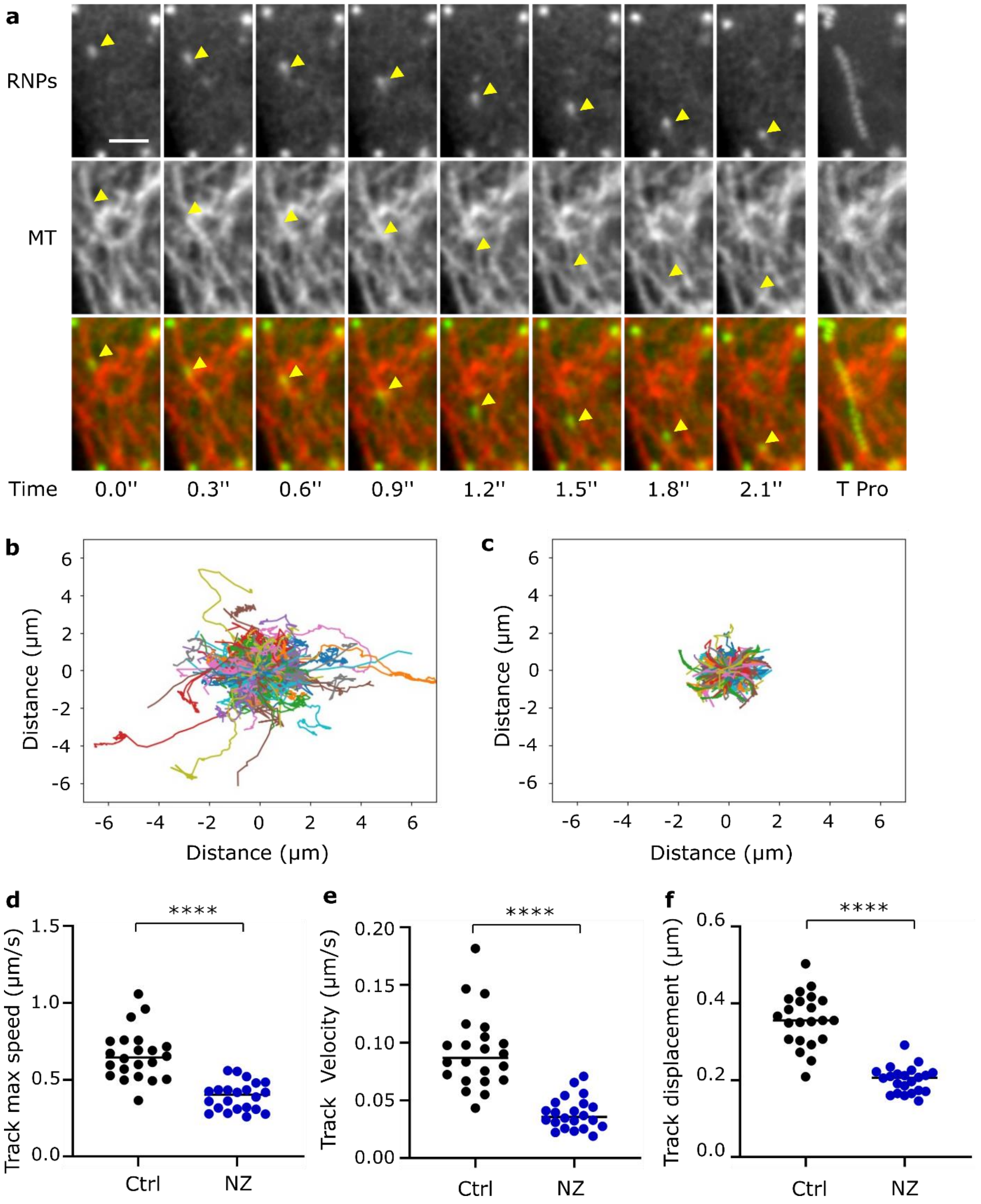

Figure 4. Fast long range RNPs motions are dependent of microtubules network. a) Time series of live images of RNPs (in green) moving along MT stained by fluorescent docetaxel-647 (in red) in RSV-GFP-N infected A549 cells. Images stacks (2 z-steps) were processed as maximum projections and visualized after gaussian filter fixed at 0.5. Yellow arrows point positions of a moving RNP. The last image shows a time projection (T Pro). Scale bar 2µm. **b to f)** RSV-GFP-N infected HEp-2 cells were treated at 17 h.p.i. with nocodazole (10µM, NZ) or DMSO (Ctrl) for 1 h before live-imaging and track analysis. **b, c)** Centered projection of the tracks of RNPs in a mock (c) or a nocodazole (d) treated cell analyzed during 60s. Representative images. **d, e, f)** Each data point represents the median of track maximum speed, track velocity and track displacement from an individual cells. **** *p*< 0.0001 using t test with Welch's correction. Data are from 22 cells (DMSO) and 23 cells (NZ) from 3 independent experiments.

Rab11 and RNPs colocalize in infected cells.

Recycling endosome vesicles, marked by the small GTPase Rab11, are known to be involved in RNPs trafficking of several negative strand RNA viruses(25,26,28,34). To investigate the recycling endosomes involvement in RSV RNPs trafficking, we analyzed Rab11a and RSV RNPs intracellular localizations. We infected A549 cells constitutively expressing HA-Rab11a (A549-HA-Rab11a) with wild type RSV (35). At the indicated time points, cells were fixed and N and HA-Rab11a were detected by immunostaining. Both N and HA staining revealed numerous individual cytoplasmic dots corresponding respectively to vRNPs and Rab11a positive endosomes, which exhibited partial colocalization (Fig 5). To quantify the colocalisation between Rab11a and N positives dots, we extracted spot detections and performed object-based colocalization analysis using Icy software. This method first segments the spots and then analyzes their relative distribution with second order statistics enabling robust quantification of colocalized objects (36). This analysis revealed that approximately 30% of N spots colocalize with Rab11a positive vesicles at 12 to 18h p.i. with a slight decrease at 24h p.i.. In contrast, less than 10% of N spots colocalize with EEA1, a marker of early endosomes used as a negative control. Similar results were obtained in A549 and HEp-2 cells (Fig S4). These data suggest an involvement of Rab11a in vRNPs traffic.

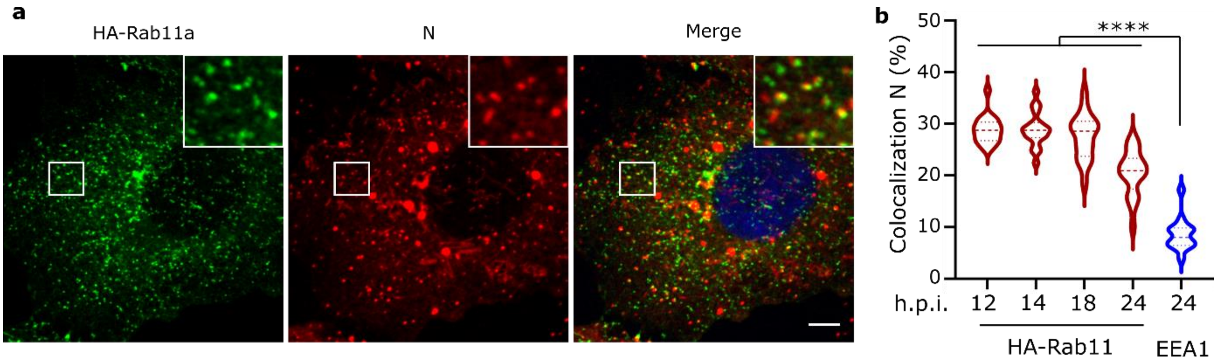

Figure 5 : Colocalization of RNPs and Rab11a in RSV infected cells. a) A549-HA-Rab11a cells infected with RSV for 18 h. Rab11a (green) and RNPs (Red) were detected by immunostaining of HA and N. Images were visualized after gaussian filter fixed at 0.5. Scale bar 5 µm. The boxed areas enclose Rab11a and N spots shown magnified. **b)** Percentage of N spots colocalizing with HA-Rab11a or EEA1 positive spots calculated using Icy Software. Violin plots show the median (center line) and the first and third quartiles (upper and lower hinges). Results are from 70 cells from 3 independent experiments. **** p<0.0001 using Brown-Forsythe and Welch's ANOVA test followed by Dunn's test for multiple comparisons.

Rab11 pathway is involved in RNPs fast and directed movements

To determine if Rab11a could be involved in RNPs trafficking, we set up an experiment to visualize both RNP and Rab11 movements in living infected cells. A549 and HEp-2 cells were infected with RSV-GFP-N and transiently transfected with a mCherry-Rab11a expression vector. At 18-20h.p.i., we performed

dual color time-lapse imaging to assess the dynamic of both Rab11a and RNPs. Interestingly, we observed fast-moving RNPs moving together with Rab11a positive vesicles demonstrating that at least some of the fast-moving RNPs are associated with Rab11a positive structures (Fig 6a and Supplementary Movie 8). Strikingly, RNPs appeared as pulled up by the Rab11 positive spots. RNPs associated with Rab11 positive vesicles were no longer mobile when cells were treated with nocodazole suggesting that these movements were microtubule-dependent (Supplementary Movie 9). The dual color acquisition settings unfortunately prevent automatic tracking of RNPs and Rab11a in this experiment.

To further demonstrate the essential role of Rab11 in RNPs export, we performed SiRNA depletion experiments. A549 cells were transiently transfected with small interfering RNA (SiRNA) targeting Rab11a or control scrambled SiRNA for 48h and subsequently infected with RSV-GFP-N for 18-20h before live imaging. Western blot analysis of the whole cell lysates using an antibody against Rab11a showed a considerable decrease in Rab11a levels in SiRNA targeting Rab11a treated cells compared to the control (Fig. S4c). RNPs movements were quantified as described above. The track velocity, track maximum velocity and track displacement were significantly reduced in Rab11a silenced cells as compared to the control (Fig 6, Supplementary Movies 10 and 11). The centered projection of the tracks from one cell illustrates the reduction of track displacement. These results suggest that Rab11a is involved in RNPs trafficking.

Since our data indicate that RSV hijacks the Rab11a pathway for RNP export, we examined whether RSV infection impacted in turn ER function. To this end, we explored transferrin recycling, which is dependent on ER functioning. Mock or RSV infected A549 cells were incubated in the presence of fluorescent transferrin and were then placed in fresh medium. Cytoplasmic fluorescence (corresponding to non-recycled transferrin) was quantified after 0 and 20 min of incubation in fresh medium (Fig S5). Interestingly, the infected cells exhibit an increase in transferrin recycling as compared to uninfected ones suggesting an acceleration of the traffic of RE. Altogether, these data suggest that RSV not only uses the recycling endosome pathway but also manipulates its functioning.

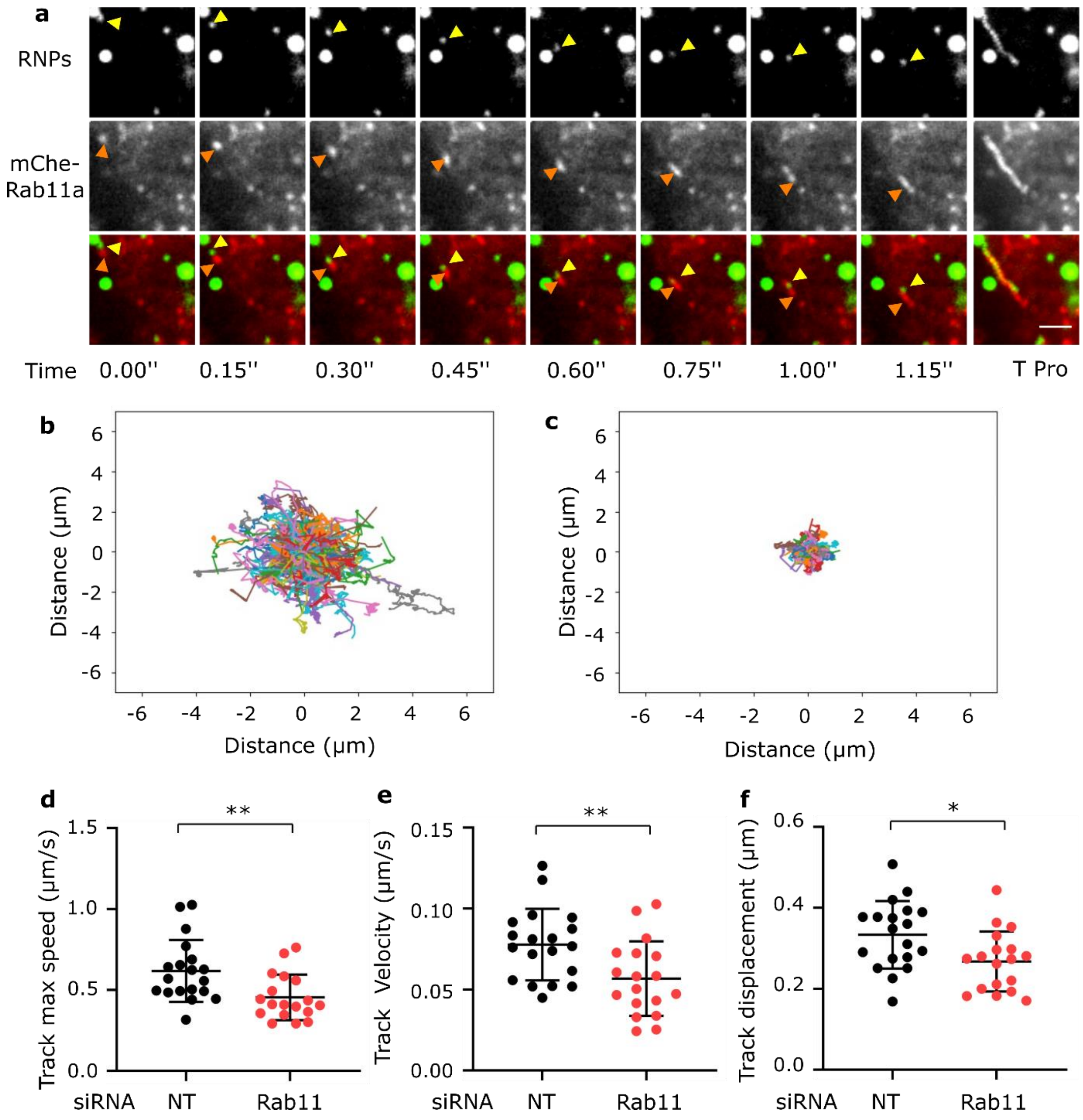

Figure 6: Rab11 is involved in fast and directed RNPs movements. a) Time series of live images of A549 cells transiently expressing mCherry-Rab11a (red) and infected for 18h with RSV-GFP-N (in green). Images were visualized after gaussian filter fixed at 0.5. Positions of a RNP moving together with a mCherry-Rab11a positive object are pointed out by yellow (RNP) and orange (Rab11a) arrow heads. The last image shows a time projection (T Pro). Scale bar 2µm. **b to f)** A549 cells were transiently transfected with siRNA targeting Rab11a or non targeting ones (NT) for 48h then infected with RSV-GFP-N for 18 h before live-imaging and track analysis. **b, c)** Centered projection of the tracks of RNPs in a NT (c) or Rab11a (d) KO cells analyzed during 60s. Representative images. **d, e and f)** Each data point represents the median of track maximum speed, track velocity and track displacement from an individual cells. ** $p< 0.01$, $p<0.05$ using t test with Welch's correction. Data are from 19 cells (NT) and 18 cells (Rab11a) from 3 independent experiments.

Biochemical validation of RNP-Rab11a association

We further assessed the association between RNPs and Rab11a performing co-immunoprecipitation (IP) analysis. A549 cells stably expressing HA-Rab11a were infected for 16h with RSV-GFP-N or RSV expressing GFP (RSV-GFP) as a control. Cell lysates were incubated with beads coated with anti-GFP antibodies and IP was carried on. Both pre-purification and IP samples were analyzed by western blot to reveal N, P and HA-Rab11 (Fig 7). Wild type N and P proteins were efficiently purified together with the GFP-N protein suggesting that the whole RNP is co-immunoprecipitated with GFP-N. The HA-Rab11a protein was also repeatedly co-immunoprecipitated with the GFP-N when no corresponding band was visible in the control. Consistent with the colocalisation and trafficking experiments, only a small fraction of Rab11a was co-immunoprecipitated with the RNPs. The mirror IP was performed on A549-HA-Rab11 cells infected with wild type RSV for 16h using anti-HA antibodies. N protein was found in the IP fraction of Rab11HA expressing cells but not of wild type A549 cells used as control. Interestingly P was also co-immunoprecipitated with HA-Rab11a suggesting that Rab11 is interacting with the RNP. To further investigate this point, we performed similar IP experiments on cells infected with a recombinant RSV expressing a L protein fused to GFP (RSV-L-GFP). The GFP is inserted in the hinge 2 region of L (11). Western blot analysis of the IP fraction failed to reveal the GFP-L most likely due to a lack of sensitivity and poor transfer of a large protein like L. However, N and P were co-immunoprecipitated thus validating the IP and confirming the capture of the RNP together with the GFP-L. Finally, we detected HA-Rab11a repeatedly and specifically in the IP sample consistent with an interaction between Rab11a and the RNP.

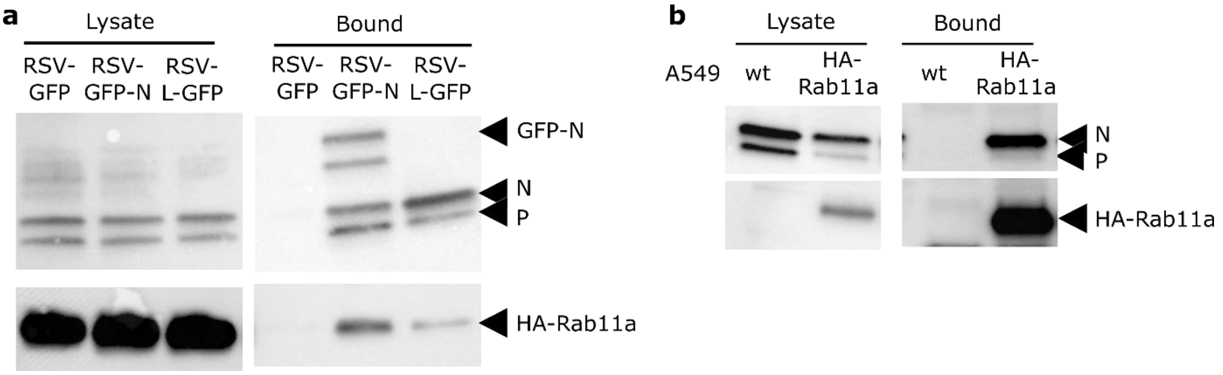

Figure 7: Co-immunoprecipitation of Rab11a and RSV RNPs in infected cells. (a) A549 stably expressing HA-Rab11a were infected with RSV-GFP, RSV-GFP-N or RSV-L-GFP as indicated for 16h, lysed and incubated with beads against GFP. Cell Lysates and bound fractions were analyzed by western-blot using rabbit polyclonal anti-N, anti-P and anti-HA antibodies. One representative experiment out of 3 (RSV-GFP-N) or 2 (RSV-L-GFP) is shown. **(b)** A549 WT or stably expressing HA-Rab11a as indicated were infected with RSV for 16h, lysed and incubated with beads against HA. Western blot analysis of the co-immunoprecipitated proteins is shown. One representative experiment out of 2.

Discussion

The understanding of many aspects of RSV multiplication has progressed considerably in recent years. However, the late stages of the viral cycle remain poorly understood. In particular, the mechanisms of transport of RSV RNPs from cytoplasmic viral factories to viral assembly sites at or near the plasma membrane remain unexplored. In this study we were able to observe for the first time the movement of RNPs in infected cells using a recombinant RSV. Our strategy was to engineer a recombinant virus expressing an N protein fused to a fluorescent tag to produce fluorescent RNPs. To our knowledge this is the first description of a RSV expressing a tagged N. Expression of this additional GFP-N does not deeply alter the replicative capacities of the virus. Noteworthy, fluorescent N perfectly colocalizes with the wild type N in the viral structures (IBs, RNPs and viral filaments) strongly suggesting that GFP-N is indeed incorporated in RSV RNPs. These data validate the use of the RSV-GFP-N as a new tool to study trafficking of RSV RNPs. In theory, more than 2000 N monomers are required to encapsidate the RSV 15kb genomic RNA (10). Due to the transcription gradient, GFP-N is expected to be less abundant than the wild-type N. Nonetheless, the oligomerization of N and GFP-N on the viral RNA leads to an amplification of the fluorescent signal and allows for the detection of vRNPs. The GFP-N virus thus represents a tool of choice for the monitoring of vRNPs in living cells. An alternative strategy based on the labeling of the viral genome with nucleotide probes has been used to analyze the dynamics of RNPs during entry or the movement of viral filaments at the cell surface. However, this method did not enable RNPs trafficking analysis (12,13). vRNPs are small, highly mobile objects. To track them, we had to find a compromise between the number of optical slices (z) analyzed and the time interval between 2 images. Our optimized pipeline relies on the acquisition of images on 2 to 3 optical planes with a total thickness of 0.7 to 2.3 µm and time-frame intervals of 0.07 to 0.21s. Our experiments were conducted on HEp-2 or A549 cells which are very thin as compared to some other cell types. Expanding the method to other cells is likely to require further optimization. In these cells, these parameters allowed a proper automatic tracking of mobile RNPs that are visible in the analyzed volume on several successive images. The potential of our method allowed us to generate high-throughput live imaging datasets otherwise inaccessible with more classical approaches. We were able to track hundreds of particles per cell and provide a robust characterization of vRNPs movements in different conditions. A large proportion of the observed vRNPs appeared immobile or animated by low amplitude "Brownian movements". The resolution of our microscopy methods (around 250nm) does not allow fine characterization of very low amplitude non-directional movements. In constrat, during the 60s acquisitions we performed, we observed that about 10% of the vRNPs were animated by fast and directional movements." These are believed to be the ones actively trafficking. The characteristics of

these motions, in particular the maximum speed above 1-2 µm/s are in themselves suggestive of MT-related transport (32).

The importance of the microtubule network in RSV multiplication has already been established by previous studies. Treatment of infected cells with nocodazole was shown to reduce virion production by about 1 log with no indication of which stage of multiplication was disrupted (22,33). Here, our results clearly establish that vRNPs are transported rapidly and over long distances on the MT network. Indeed, we observed vRNPs moving on MTs and were able to verify the almost complete disappearance of these movements upon disassembly of the MT network. Many negative RNA viruses also hijack the MT network and its associated motors for the transport of their RNPs. For example, RNPs from Influenza A , Sendai or measles viruses move along this network(25,26,37–39). As what has been observed for RSV, depolymerization of the MT network does not completely abolish the replication of these viruses (26,40,41). For influenza viruses, conflicting results were reported on the effect of nocodazole treatment on viral multiplication (42) or on movement of vRNPs (37). These data suggest that alternate pathways exist for the transport and/or spread of these viruses. It is worth noting that our study, like other published studies, was performed on epithelial cell lines, and not on differentiated polarized cells, which are the *in vivo* target cells of these viruses are. In such cells cytoskeleton organization is different and the extent of MT dependent transportation and alternative transport pathways remains to be explored.

Our data clearly demonstrate that the fast and long-distance transport of RSV RNPs is dependent on the recycling endosome. Inhibition of Rab11a expression indeed significantly reduces the travel length and the speed of vRNPs. However, vRNP transport is not completely suspended, suggesting either a partial inhibition of Rab11a expression, an involvement of Rab11b and/or the existence of alternative transport pathways. Direct observation of joint movements of Rab11a with vRNPs in living cells clearly demonstrate the involvement of ERC in vRNPs transport. Our videos show vRNPs literally pulled by the Rab11a positive structures. The immunoprecipitation experiments confirm an association between vRNPs and Rab11a although not demonstrative of a direct interaction. At a given time point about 30% of vRNPs are colocalized with Rab11a suggesting that the association of vRNPs with Rab11a is transient. In line with this observation, only a fraction of Rab11a and vRNPs are co-immunoprecipitated and only a small proportion of vRNPs are engaged in fast movement. Rab11 is able to engage motors linked to either microtubule or actin networks. The movements of Rab11a-associated vRNPs we observed, are fast and abolished by MT depolymerization demonstrating that they are MT dependent. Overall, our data support the following model for RSV RNPs export: 1) vRNPs transiently associate with Rab11a-positive vesicles 2) these Rab11a-positive vesicles transport vRNPs rapidly and over long distances along the microtubule network. Moreover, RSV not only diverts the Rab11 pathway for its RNPs

transport but also manipulates it. Indeed, we observed that RSV infection increases transferrin recycling, consistent with previous analysis of Rab11a vesicles motion that revealed increase in velocity and in high distance displacement in RSV infected cells (38).

In recent years Rab11 has emerged as a key cellular factor involved in various stages of the multiplication of several viruses. Some viruses enter the cell using the ERC as suggested by the association of HHV8 or Dengue viral capsids with Rab11 endosomes soon after infection (43,44). Furthermore, it has previously been shown that effectors of the Rab11 pathway: FIP1, FIP2 and MyosinVb support RSV release at the apical side of the membrane. FIP2 is even thought to regulate the length of viral filaments (45,46) suggesting an involvement of the ERC in the budding steps of RSV. Rab11 and its partner FIP3 have also been implicated in the budding of influenza viruses (47) and recent results show that the integrity of the Rab11 pathway is required for Ebola virus VP40 transfer to the membrane and for VLP release (48). Subversion of Rab11 pathway for the transport of the newly synthetized RNPs of negative RNA viruses to plasma membrane is extensively documented. Previous studies showed that newly synthesized RNPs from Measles, Mumps, Influenza, Parainfluenza 1, Sendai viruses or a new world Hantavirus are associated with Rab11 positive endosomes and that Rab11 is involved in their transfer to the plasma membrane (26–28,39–41,47,49–51). However, the precise mechanisms of vRNPs associations with Rab11 and the pathways involved in transport remain to be defined. The numerous studies of the export of Influenza virus RNPs have shown that vRNPs recruitment depends on a direct protein-protein interaction between a unit of the viral polymerase complex PB2 and the active form of Rab11a, and that the Kinesin Kif13a is one of the motors involved in their transport to the membrane (39,41,52). The L polymerase of Sendai virus has also been shown to be involved in the recruitment of vRNPs to ERC vesicles (53). Rab11a is co-immunoprecipitated with RSV L suggesting that the RSV polymerase could similarly promote Rab11a-vRNP association. However, since the whole vRNP is captured in our experiments, further investigations are needed to determine if L is critical for recruiting Rab11.

In conclusion, our results establish that RSV RNPs associated with Rab11a vesicles are transported along MTs. These data are a first step forward in the understanding of vRNP export. They pave the way for further work aimed at identifying the viral proteins, the Rab11 cellular partners and the molecular motors involved in vRNPs transport. In the future, interactions between RSV and the ER pathway could represent a new therapeutic target to block the late stages of the RSV replication cycle.

Methods

Cells

HEp-2 cells (ATCC, CCL-23) and A549 cells (ATCC, CCL-185) were maintained in Eagle's minimum essential medium (MEM) and in Dulbecco modified essential medium (DMEM), respectively, supplemented with 10% heat-inactivated fetal bovine serum (FBS) supplemented with penicillin–streptomycin solution. A549 cells constitutively expressing the HA-Rab11a (WT) are a kind gift from Dr N Naffakh (35) and were grown in DMEM supplemented with 1µg/mL puromycin. Cells were grown in an incubator at 37 °C in 5% CO_2. HEp-2 cells and A549 tested negative for mycoplasma using a MycoAlert PLUS Mycoplasma Detection Kit (Lonza).

Virus and plasmids

All the viral sequences were derived from the human RSV strain Long, ATCC VR-26. The wild-type RSV, the RSV-GFP, the RSV-GFP-N and the RSV-L-GFP were rescued by reverse genetics as previously described (30,54). To construct the reverse genetic vector for RSV-GFP-N, the GFP-N coding sequence was amplified from the pmGFP-N vector (11) by PCR using specific primers (sequence available upon request) containing RSV gene start and gene end sequences and was cloned into a Eco105I restriction site between the RSV M and SH genes in the pACNR-rHRSV (30). The nucleotide sequence of RSV-GFP-N was deposited in the Genbank nucleotide database with accession code OM326756. pmCherry-Rab11a was constructed by inserting the Cherry coding sequence in peGFP-Rab11a (55) (kind gift from Dr Sauvonnet) between NheI and XhoI restriction sites. All constructs were verified by sequencing. Experiments were performed with viral stock amplified on HEp-2 cells at 37 °C after three to five passages. Plaque assay were performed at 37 °C on HEp-2 cells using Avicel overlay as previously described (30).

Antibodies and reagents

The rabbit polyclonal anti-P and anti-N were obtained by repeated injection of purified recombinant protein produced in *Escherichia coli* as previously described (56). The rabbit anti-Rab11a is from Thermofisher, the rabbit anti-EEA1 and rabbit anti-HA from Cell signaling, the rat anti-HA is from Roche, and the mouse anti-F was from Abcam. The mouse monoclonal anti-P 021/2P (57) is a kind gift from Dr JF Eléouët. Secondary antibodies raised against mouse or rabbit IgG (H+L) and conjugated to Alexa Fluor 488, 594 or 647 were from Thermofisher. Secondary antibodies raised against mouse or rabbit IgG (H+L) and conjugated to horseradish peroxidase were from Promega. DMSO, Nocodazole and Cytochalasin D were from Sigma. Live staining of Microtubules was achieved with SiR-tubulin Kit 647 as described by the manufacturer (Spirochrome).

Cell infection and transfection

All infections were performed at 37 °C on exponentially growing HEp-2 or A549 cells at a multiplicity of infection of 1 (except indicated otherwise). For Rab11cherry and GFP-N live imaging, A549 or HEp-2 cells were seeded at 90% confluence in Ibidi µ-Slide Angiogenesis (Ibidi) and were transfected with 0.5µg of pmCherry-Rab11a (per 0.5 10^6 cells) using Lipofectamine 2000 (Invitrogen) according to the manufacturer recommendations. Then cells were infected with RSV-GFP-N 1h post-transfection. SiRNA transfections were performed on A549 cells newly seeded on Ibidi µ-Slide Angiogenesis. Briefly, a pool of 4 different siRNA targeting the same gene Rab11a (ON-TARGETplus SMARTpool, Dharmacon) or a non targeting pool was transfected simultaneously to the cell seeding, using LipoRNAiMax (Thermofisher) transfection reagent. 0.3 µL of LipoRNAiMax per well (per 0.2.10^5 cells) were used with a final siRNA concentration of 10 nM. Cells were incubated at 37°C for 48h before being infected.

Immunofluorescence – Colocalization analysis

Cells were grown on glass coverslips in 24 wells plates. After infection at high MOI for the indicated times, cells were fixed with PBS-formaldehyde 4% (v/v) for 10 min, washed with PBS and permeabilized with PBS-BSA 1% (w/v)-Triton X-100 0.25% (v/v) for 10 min. Cells were incubated for 1 h in PBS-BSA 1% (w/v) with the appropriate primary antibodies, rinsed with PBS then incubated for 30 min with the appropriate Alexa Fluor-conjugated secondary antibodies and with Hoechst 33342 (1 µg/ml). Coverslips were rinsed in PBS, then mounted in ProLong diamond antifade reagent (Thermofisher). Cells were examined by confocal microscopy under a WLL Leica SP8 microscope except indicated otherwise. Representative pictures were taken.

Colocalization analysis were performed using the open-source software Icy (http://icy.bioimageanalysis.org/) (58). We used Spot Detector plugin to automatically detect objects corresponding to RNP and vesicles (Rab11a or EEA1) and then Colocalization Studio plugin (method: object based and statistical colocalisation, SODA (36), to quantify the number of RNP at a distance less than 270 nm from the cellular vesicles.

Transferrin uptake quantification

A549 cells starved for 30 min in serum-free medium containing 0.5 % (w/v) BSA were pulsed for 10 min with Tf-AF-647 (25 µg/mL, Thermofisher). Cells were then quickly incubated with cold acid buffer (0,5 M Glycine, pH 2.2) to dissociate surface-bound Tf, extensively washed with PBS, and incubated at 37 °C in complete media for 0 or 20 min before fixation with 4 % paraformaldehyde. Cells were imaged by confocal microscopy under a WLL Leica SP8 microscope. Cytoplasmic AF647 fluorescence signal was quantified using the “Measure” function of imageJ/Fiji on average z-projections. Signal intensities at 20 min were normalized to their respective mean at 0 min.

Co-Immunoprecipitation

WT or HA-Rab11a expressing A549 cells infected with RSV-GFP-N, RSV-L-GFP, RSV-GFP or RSV-WT for 16 hours were lysed in a co-IP lysis buffer ((Tris 25mMpH 7.2; NaCl 150 mM; IGEPAL® CA-630 0.2% (Sigma); glycerol 10% (v/v); EDTA 0.5mM; antiprotease and phosphatase (Thermofisher)), then incubated overnight at 4 °C with GFP-Trap beads (Chromotek) or Magnetic-HA beads (Thermofisher) according to the manufacturer's recommendations. Beads were rinsed three times in a co-IP dilution buffer (Tris 25mM pH 7.2; NaCl 150mM), then eluted in Laemmli buffer at 95 °C and analyzed by SDS-PAGE.

Live Imaging

Live imaging was performed on A549 or HEp-2 cells seeded on Ibidi µ-Slide Angiogenesis, infected and treated as described above. One-minute time-lapse acquisitions were performed at 37°C and 5% CO_2 using an inverted confocal microscope with a 100x oil-immersion lens (Plan-APOCHROMAT), a CSU-X1 spinning-disk head (Yokogawa, Japan) and a sCMOSPRIME 95B (Photometrics) camera. The whole setup was driven with MetaMorph software (Molecular Devices, Sunnyvale, CA, USA). One to three z sections with a step of 0.8 µm were acquired at 70 to 210 ms intervals for 1 min. Laser intensity was set between 10-20% power, and acquisition time was 50 ms. The raw data were processed using ImageJ software (National Institutes of Health, Bethesda, MD, USA) to perform maximum projections of z stacks images and background subtraction. Tracks analysis was performed on Imaris software 9.5.0 (Bitplane Inc.). Briefly fluorescent spots were identified using Imaris built-in spot generation algorithm (with a seeding spot size of 250 nm). Spot generation was achieved manually for each dataset using intensity at the center of each spot as the threshold. Quality threshold was fixed above 20.0. The spots were tracked over time to generate motion statistics for each cell using autoregressive motion algorithm (max distance 1µm, max gap size 2). Imaris output file "Position.csv" was further used to calculate the characteristic of each tracks (*track duration*, *track length*, *track displacement, track maximum speed*, *mean track velocity*) and to filter tracks using dedicated PYTHON Script available at GitHub (https://github.com/mawelti/RSV-RNP-TrackAnalysis). All tracks smaller than 4 steps were filtered. Coordinates of the IBs area were entered manually to remove false tracks generated by frequent false detections due to high background signal in the vicinity of the IBs (see Fig S2a,b). Tracks whose instant speed is always lower than 50% of the maximum instant speed were also filtered to remove false link between 2 slow moving objects (See Fig S2c, d, e). *Smoothed instant speed* were calculated as the ratio between the minimum distance between the first and the fourth of 4 consecutive positions and the time interval between these positions. We choose to smooth the instant speed because time interval between 2 frames being very small (0.07 to 0.21s), displacement of 250nm corresponding to position error of diffracted limited microscopy resulted in high instant speed (1.2 to

3.6 μm/s) (Fig S2 f). To compare the effect of treatments, statistical comparisons between median were performed in Prism software (Graphpad Inc.) as indicated in legends. All tests used in this report are two-sided. For visualization, the raw data were processed using ImageJ software (National Institutes of Health, Bethesda, MD, USA). Images stacks were processed as maximum projections and visualized after gaussian filter fixed at 0.5.

Acknowledgements: We thank Marie Galloux, Jean-Francois Eléouët, Nadia Naffakh and Cédric Delevoye for sharing protocols and reagents, and for helpful discussions. We are grateful to Nathalie Sauvonnet for Rab11 plasmids and helpful discussions. We thank Jennifer Risso-Ballester, Cédric Diot and Sabine Blouquit-Laye for insightful discussions and critical reading of the manuscript. We thank Aude Jobart-Malfait and Cymages platform for access to the Leica SP8 microscope and Olympus FV3000 inverted confocal microscope, which was supported by grants from the region Ile-de-France. We also acknowledge the ImagoSeine core facility of the Institut Jacques Monod and thank Xavier Baudin for support for spinning disk imaging. We acknowledge support from INSERM and Versailles Saint-Quentin University. This work was supported by ATIP-AVENIR INSERM program and the Fondation Del Duca - Institut de France.

References

1. Group Pneumonia Etiology Research for Child Health (PERCH). Causes of severe pneumonia requiring hospital admission in children without HIV infection from Africa and Asia: the PERCH multi-country case-control study. Lancet (London, England). 2019/06/27. 2019 Aug;394(10200):757–79.

2. Shi T, McAllister DA, O'Brien KL, Simoes EAF, Madhi SA, Gessner BD, et al. Global, regional, and national disease burden estimates of acute lower respiratory infections due to respiratory syncytial virus in young children in 2015: a systematic review and modelling study. Lancet (London, England). 2017 Sep;390(10098):946–58.

3. Asner S, Stephens D, Pedulla P, Richardson SE, Robinson J, Allen U. Risk factors and outcomes for respiratory syncytial virus-related infections in immunocompromised children. Pediatr Infect Dis J [Internet]. 2013;32(10):1073–6. Available from: http://www.ncbi.nlm.nih.gov/pubmed/23732222

4. Beigel JH, Nam HH, Adams PL, Krafft A, Ince WL, El-Kamary SS, et al. Advances in respiratory virus therapeutics - A meeting report from the 6th isirv Antiviral Group conference. Antiviral Res. 2019 Jul;167:45–67.

5. Shi T, Denouel A, Tietjen AK, Campbell I, Moran E, Li X, et al. Global disease burden estimates of respiratory syncytial virus-associated acute respiratory infection in older adults in 2015: A systematic review and meta-analysis. J Infect Dis. 2021;222(Xx Xxxx):S577–83.

6. Heylen E, Neyts J, Jochmans D. Drug candidates and model systems in respiratory syncytial virus antiviral drug discovery. Biochem Pharmacol [Internet]. 2017;127:1–12. Available from: http://dx.doi.org/10.1016/j.bcp.2016.09.014

7. Conley MJ, Short JM, Hutchings J, Burns AM, Streetley J, Saskia E, et al. Helical Ordering of Envelope Associated Proteins and Glycoproteins in Respiratory Syncytial Virus Filamentous Virions. 2021;

8. Collins PL, Melero JA. Progress in understanding and controlling respiratory syncytial virus: still crazy after all these years. Virus Res [Internet]. 2011 Dec [cited 2014 Feb 18];162(1–2):80–99. Available from: http://www.pubmedcentral.nih.gov/articlerender.fcgi?artid=3221877&tool=pmcentrez&rendertype=abstract

9. Noton SL, Fearns R. Initiation and regulation of paramyxovirus transcription and replication. Virology [Internet]. 2015;479–480:545–54. Available from: http://dx.doi.org/10.1016/j.virol.2015.01.014

10. Tawar RG, Duquerroy S, Vonrhein C, Varela PF, Damier-Piolle L, Castagné N, et al. Crystal structure of a nucleocapsid-like nucleoprotein-RNA complex of respiratory syncytial virus. Science [Internet]. 2009 Nov 27 [cited 2014 Feb 18];326(5957):1279–83. Available from: http://www.ncbi.nlm.nih.gov/pubmed/19965480

11. Rincheval V, Lelek M, Gault E, Bouillier C, Sitterlin D, Blouquit-Laye S, et al. Functional organization of cytoplasmic inclusion bodies in cells infected by respiratory syncytial virus. Nat Commun [Internet]. 2017 Sep;8(1):563. Available from: http://dx.doi.org/10.1038/s41467-017-00655-9

12. Santangelo PJ, Bao G. Dynamics of filamentous viral RNPs prior to egress. Nucleic Acids Res [Internet]. 2007 Jan [cited 2014 Feb 11];35(11):3602–11. Available from:

http://www.pubmedcentral.nih.gov/articlerender.fcgi?artid=1920244&tool=pmcentrez&rendertype=abstract

13. Alonas E, Lifland AW, Gudheti M, Vanover D, Jung J, Zurla C, et al. Combining single RNA sensitive probes with subdiffraction-limited and live-cell imaging enables the characterization of virus dynamics in cells. ACS Nano. 2014;8(1):302–15.

14. Kiss G, Holl JM, Williams GM, Alonas E, Vanover D, Lifland AW, et al. Structural Analysis of Respiratory Syncytial Virus Reveals the Position of M2-1 between the Matrix Protein and the Ribonucleoprotein Complex. J Virol [Internet]. 2014;88(13):7602–17. Available from: http://jvi.asm.org/cgi/doi/10.1128/JVI.00256-14

15. Shaikh FY, Cox RG, Lifland AW, Hotard AL, Williams J V., Moore ML, et al. A critical phenylalanine residue in the respiratory syncytial virus fusion protein cytoplasmic tail mediates assembly of internal viral proteins into viral filaments and particles. MBio. 2012;3(1):1–10.

16. Mitra R, Baviskar P, Duncan-Decocq RR, Patel D, Oomens AGP. The human respiratory syncytial virus matrix protein is required for maturation of viral filaments. J Virol [Internet]. 2012 Apr [cited 2014 Feb 18];86(8):4432–43. Available from: http://www.pubmedcentral.nih.gov/articlerender.fcgi?artid=3318654&tool=pmcentrez&rendertype=abstract

17. Baviskar PS, Hotard AL, Moore ML, Oomens AGP. The Respiratory Syncytial Virus Fusion Protein Targets to the Perimeter of Inclusion Bodies and Facilitates Filament Formation by a Cytoplasmic Tail -Dependent Mechanism. J Virol [Internet]. 2013 Oct [cited 2014 Feb 18];87(July):10730–41. Available from: http://www.ncbi.nlm.nih.gov/pubmed/23903836

18. Bajorek M, Galloux M, Richard C-A, Szekely O, Rosenzweig R, Sizun C, et al. Tetramerization of Phosphoprotein is Essential for Respiratory Syncytial Virus Budding while its N Terminal Region Mediates Direct Interactions with the Matrix Protein. J Virol. 2021 Jan;

19. Meshram CD, Oomens AGP. Identification of a human respiratory syncytial virus phosphoprotein domain required for virus-like-particle formation. Virology [Internet]. 2019;532(April):48–54. Available from: https://doi.org/10.1016/j.virol.2019.04.001

20. Vanover D, Smith D V, Blanchard EL, Alonas E, Kirschman JL, Lifland AW, et al. RSV glycoprotein and genomic RNA dynamics reveal filament assembly prior to the plasma membrane. Nat Commun. 2017 Sep;8(1):667.

21. Luby-Phelps K. Cytoarchitecture and physical properties of cytoplasm: volume, viscosity, diffusion, intracellular surface area. Int Rev Cytol. 2000;192:189–221.

22. Shaikh FY, Utley TJ, Craven RE, Rogers MC, Lapierre LA, Goldenring JR, et al. Respiratory syncytial virus assembles into structured filamentous virion particles independently of host cytoskeleton and related proteins. PLoS One. 2012;7(7):e40826.

23. Jeffree CE, Brown G, Aitken J, Su-Yin DY, Tan BH, Sugrue RJ. Ultrastructural analysis of the interaction between F-actin and respiratory syncytial virus during virus assembly. Virology [Internet]. 2007 Dec 20 [cited 2014 Feb 18];369(2):309–23. Available from: http://www.ncbi.nlm.nih.gov/pubmed/17825340

24. Horgan CP, McCaffrey MW. The dynamic Rab11-FIPs. Biochem Soc Trans. 2009;37(5):1032–6.

25. Nakatsu Y, Ma X, Seki F, Suzuki T, Iwasaki M, Yanagi Y, et al. Intracellular transport of the measles virus ribonucleoprotein complex is mediated by Rab11A-positive recycling endosomes and drives virus release from the apical membrane of polarized epithelial cells. J Virol [Internet].

2013;87(8):4683–93. Available from: http://www.pubmedcentral.nih.gov/articlerender.fcgi?artid=3624359&tool=pmcentrez&rendertype=abstract

26. Chambers R, Takimoto T. Trafficking of sendai virus nucleocapsids is mediated by intracellular vesicles. PLoS One [Internet]. 2010 Jan [cited 2014 Feb 15];5(6):e10994. Available from: http://www.pubmedcentral.nih.gov/articlerender.fcgi?artid=2881874&tool=pmcentrez&rendertype=abstract

27. Katoh H, Nakatsu Y, Kubota T, Sakata M, Takeda M, Kidokoro M. Mumps Virus Is Released from the Apical Surface of Polarized Epithelial Cells, and the Release Is Facilitated by a Rab11-Mediated Transport System. J Virol. 2015;89(23):12026–34.

28. Avilov S V., Moisy D, Naffakh N, Cusack S. Influenza A virus progeny vRNP trafficking in live infected cells studied with the virus-encoded fluorescently tagged PB2 protein. Vaccine [Internet]. 2012;30(51):7411–7. Available from: http://dx.doi.org/10.1016/j.vaccine.2012.09.077

29. Stenmark H. Rab GTPases as coordinators of vesicle traffic. Nat Rev Mol Cell Biol. 2009 Aug;10(8):513–25.

30. Rameix-Welti M-A, Le Goffic R, Hervé P-L, Sourimant J, Rémot A, Riffault S, et al. Visualizing the replication of respiratory syncytial virus in cells and in living mice. Nat Commun [Internet]. 2014 Jan [cited 2015 Jan 31];5:5104. Available from: http://www.ncbi.nlm.nih.gov/pubmed/25277263

31. Lifland AW, Jung J, Alonas E, Zurla C, Crowe JE, Santangelo PJ. Human Respiratory Syncytial Virus Nucleoprotein and Inclusion Bodies Antagonize the Innate Immune Response Mediated by MDA5 and MAVS. J Virol. 2012;

32. Greber UF, Way M. A superhighway to virus infection. Cell. 2006 Feb;124(4):741–54.

33. Kallewaard NL, Bowen AL, Crowe JE. Cooperativity of actin and microtubule elements during replication of respiratory syncytial virus. Virology [Internet]. 2005 Jan 5 [cited 2014 Feb 18];331(1):73–81. Available from: http://www.ncbi.nlm.nih.gov/pubmed/15582654

34. Katoh H, Nakatsu Y, Kubota T, Sakata M, Takeda M, Kidokoro M. Mumps Virus Is Released from the Apical Surface of Polarized Epithelial Cells, and the Release Is Facilitated by a Rab11-Mediated Transport System. J Virol. 2015;89(23):12026–34.

35. De Castro Martin IF, Fournier G, Sachse M, Pizarro-Cerda J, Risco C, Naffakh N. Influenza virus genome reaches the plasma membrane via a modified endoplasmic reticulum and Rab11-dependent vesicles. Nat Commun [Internet]. 2017;8(1). Available from: http://dx.doi.org/10.1038/s41467-017-01557-6

36. Lagache T, Grassart A, Dallongeville S, Faklaris O, Sauvonnet N, Dufour A, et al. Mapping molecular assemblies with fluorescence microscopy and object-based spatial statistics. Nat Commun [Internet]. 2018;9(1):102–8. Available from: http://dx.doi.org/10.1038/s41467-018-03053-x

37. Avilov S V, Moisy D, Munier S, Schraidt O, Naffakh N, Cusack S. Replication-competent influenza A virus that encodes a split-green fluorescent protein-tagged PB2 polymerase subunit allows live-cell imaging of the virus life cycle. J Virol [Internet]. 2012 Feb [cited 2014 Feb 18];86(3):1433–48. Available from: http://www.pubmedcentral.nih.gov/articlerender.fcgi?artid=3264389&tool=pmcentrez&rendertype=abstract

38. Bhagwat AR, Le Sage V, Nturibi E, Kulej K, Jones J, Guo M, et al. Quantitative live cell imaging reveals influenza virus manipulation of Rab11A transport through reduced dynein association. Nat Commun [Internet]. 2020;11(1):1–14. Available from: http://dx.doi.org/10.1038/s41467-019-13838-3

39. Momose F, Sekimoto T, Ohkura T, Jo S, Kawaguchi A, Nagata K, et al. Apical Transport of Influenza A Virus Ribonucleoprotein Requires Rab11-positive Recycling Endosome. PLoS One [Internet]. 2011;6(6):e21123. Available from: http://dx.plos.org/10.1371/journal.pone.0021123

40. Nakatsu Y, Ma X, Seki F, Suzuki T, Iwasaki M, Yanagi Y, et al. Intracellular Transport of the Measles Virus Ribonucleoprotein Complex Is Mediated by Rab11A-Positive Recycling Endosomes and Drives Virus Release from the Apical Membrane of Polarized Epithelial Cells. J Virol. 2013;87(8):4683–93.

41. Amorim MJ, Bruce EA, Read EKC, Foeglein A, Mahen R, Stuart AD, et al. A Rab11- and microtubule-dependent mechanism for cytoplasmic transport of influenza A virus viral RNA. J Virol [Internet]. 2011;85(9):4143–56. Available from: http://www.pubmedcentral.nih.gov/articlerender.fcgi?artid=3126276&tool=pmcentrez&rendertype=abstract

42. Nturibi E, Bhagwat AR, Coburn S, Myerburg MM, Lakdawala SS. Intracellular Colocalization of Influenza Viral RNA and Rab11A Is Dependent upon Microtubule Filaments. J Virol. 2017;91(19):1–20.

43. Acosta EG, Castilla V, Damonte EB. Differential requirements in endocytic trafficking for penetration of dengue virus. PLoS One. 2012;7(9):e44835.

44. Greene W, Gao S-J. Actin dynamics regulate multiple endosomal steps during Kaposi's sarcoma-associated herpesvirus entry and trafficking in endothelial cells. PLoS Pathog. 2009 Jul;5(7):e1000512.

45. Brock SC, Goldenring JR, Crowe JE. Apical recycling systems regulate directional budding of respiratory syncytial virus from polarized epithelial cells. Proc Natl Acad Sci U S A. 2003;100(25):15143–8.

46. Utley TJ, Ducharme N a, Varthakavi V, Shepherd BE, Santangelo PJ, Lindquist ME, et al. Respiratory syncytial virus uses a Vps4-independent budding mechanism controlled by Rab11-FIP2. Proc Natl Acad Sci U S A. 2008;105(29):10209–14.

47. Bruce EA, Digard P, Stuart AD. The Rab11 Pathway Is Required for Influenza A Virus Budding and Filament Formation. J Virol. 2010;84(12):5848–59.

48. Nanbo A, Ohba Y. Budding of Ebola Virus Particles Requires the Rab11-Dependent Endocytic Recycling Pathway. J Infect Dis. 2018;218(Suppl 5):S388–96.

49. Stone R, Hayashi T, Bajimaya S, Hodges E, Takimoto T. Critical role of Rab11a-mediated recycling endosomes in the assembly of type I parainfluenza viruses. Virology [Internet]. 2016;487:11–8. Available from: http://dx.doi.org/10.1016/j.virol.2015.10.008

50. Eisfeld AJ, Kawakami E, Watanabe T, Neumann G, Kawaoka Y. RAB11A is essential for transport of the influenza virus genome to the plasma membrane. J Virol [Internet]. 2011;85(13):6117–26. Available from: http://www.pubmedcentral.nih.gov/articlerender.fcgi?artid=3126513&tool=pmcentrez&rendertype=abstract

51. Rowe RK, Suszko JW, Pekosz A. Roles for the recycling endosome, Rab8, and Rab11 in hantavirus release from epithelial cells. Virology [Internet]. 2008;382(2):239–49. Available from: http://dx.doi.org/10.1016/j.virol.2008.09.021

52. Ramos-Nascimento A, Kellen B, Ferreira F, Alenquer M, Vale-Costa S, Raposo G, et al. KIF13A mediates trafficking of influenza Avirus ribonucleoproteins. J Cell Sci. 2017;130(23):4038–50.

53. Genoyer E, Kulej K, Hung CT, Thibault PA, Azarm K, Takimoto T, et al. The viral polymerase complex mediates the interaction of viral ribonucleoprotein complexes with recycling endosomes during sendai virus assembly. MBio. 2020;11(4):1–20.

54. Bouillier C, Rincheval V, Sitterlin D, Blouquit-Laye S, Desquesnes A, Eléouët J-F, et al. Generation, Amplification, and Titration of Recombinant Respiratory Syncytial Viruses. J Vis Exp. 2019 Apr;(146):e59218.

55. Mounier J, Boncompain G, Senerovic L, Lagache T, Chrétien F, Perez F, et al. Shigella effector IpaB-induced cholesterol relocation disrupts the Golgi complex and recycling network to inhibit host cell secretion. Cell Host Microbe. 2012 Sep;12(3):381–9.

56. Castagne N. Biochemical characterization of the respiratory syncytial virus P-P and P-N protein complexes and localization of the P protein oligomerization domain. J Gen Virol [Internet]. 2004 Jun 1 [cited 2014 Feb 18];85(6):1643–53. Available from: http://vir.sgmjournals.org/cgi/doi/10.1099/vir.0.79830-0

57. Garcia J, Garcia-Barreno B, Vivo A, Melero JA. Cytoplasmic inclusions of respiratory syncytial virus-infected cells: formation of inclusion bodies in transfected cells that coexpress the nucleoprotein, the phosphoprotein, and the 22K protein. Virology. 1993 Jul;Jul;195(1)(1):243–7.

58. de Chaumont F, Dallongeville S, Chenouard N, Hervé N, Pop S, Provoost T, et al. Icy: an open bioimage informatics platform for extended reproducible research. Nat Methods. 2012 Jun;9(7):690–6.

Click here to access/download
Supporting Information
Cosentino_Supplementary figures and movies legends.docx

video 1

Click here to access/download
Supporting Information
Supplementary movie S1 (6fps).avi

Click here to access/download
Supporting Information
Supplementary movie S2 (6fps)small.mp4

video 3

Click here to access/download
Supporting Information
Supplementary movie S3 (8fps).avi

video 4

Click here to access/download
Supporting Information
Supplementary movie S4 (8fps).avi

video 5

Click here to access/download

Supporting Information

Supplementary movie S5 (8fps).avi

video 6

Click here to access/download

Supporting Information

Supplementary movie S6 (12fps).avi

video 7

Click here to access/download
Supporting Information
Supplementary movie S7 (12fps).avi

video 8

Click here to access/download
Supporting Information
Supplementary movie S8 (8fps).avi

video 9

Click here to access/download

Supporting Information

Supplementary movie S9 (8fps).avi

video 10

Click here to access/download
Supporting Information
Supplementary movie S10 (12fps).avi

video 11

Click here to access/download
Supporting Information
Supplementary movie S11 (12fps).avi
