# Supplementary material for: Respiratory syncytial virus ribonucleoproteins hijack microtubule Rab11 dependent transport for intracellular trafficking
Source: PLoS Pathog. 2022 Jul 7;18(7):e1010619. doi: 10.1371/journal.ppat.1010619 (PMC9262236; doi:10.1371/journal.ppat.1010619)
Supplement: Attachment — Submitted filename: Point-by-point response_Cosentino et al.docx [file ppat.1010619.s018.docx]

# Point by response to reviewers’ comments.

First, we thank the reviewers for their constructive suggestions that allowed us to improve the manuscript. In the following we provide a point-by-point response to the points raised by the referees.

## Reviewer's Responses to Questions

## Part I - Summary Please use this section to discuss strengths/weaknesses of study, novelty/significance, general execution and scholarship.

Reviewer #1: The authors have generated a recombinant RSV with a second copy of the N (nucleocapsid) protein with GFP fused to its N-terminus, inserted further down the genome so that it would be produced in lower amounts than the unmodified N. This modified RSV replicates as well as its parent and can be visualized in infected cells in culture. They have found that Rab11b co-localizes with the nucleocapsid and is co-transported along microtubules within live cells. Nocodazole, a disruptor of microtubules, inhibits, but does not completely halt this transport. The results illustrate rapid transport of RSV nucleocapsids through cells using the recycling endosome mechanism.

Reviewer #2: Cosentino et al present a well-written and well-executed paper that demonstrates that RSV utilizes microtubules and Rab-11 for transport of vRNPs within the cytoplasm. A method was developed to track vRNPs in live cells. Measurements to track speed and distance are necessarily somewhat arbitrary but are sufficient to support the main findings. The movies are both supportive of their claims and beautiful to watch. Previous papers have demonstrated the importance of microtubules for RSV replication and the effects of nocodazole, but the present work makes a first step to begin to understand the mechanism. Some of the bigger questions also posed by the authors, how and whether vRNPs move from factory sites to the membrane, and the role of inclusion bodies therein, remains unexplored and undiscussed. This somewhat lessens the impact of the paper, as it not surprising that RSV, like many other negative-strand RNA viruses, uses microtubules and rab11.

We agree with the referee. While our data contribute to a better understanding of the late stages of the RSV multiplication cycle, many questions remain. We have enriched the discussion on the points raised by the referee but further studies are needed to gain a full understanding of the mechanisms.

## Part II – Major Issues: Key Experiments Required for Acceptance Please use this section to detail the key new experiments or modifications of existing experiments that should be absolutely required to validate study conclusions.

Reviewer #1: The experiments are very thorough, with multiple approaches and assays to demonstrate this interaction, transport and quantify its speed in several ways. The results are clear and convincing.

We thank the reviewer 1 for its positive assessment of our work

Reviewer #2: Whereas the demonstration of vRNP movement within the cytoplasm and involvement of Rab11 is a step forward in understanding, the role of inclusion bodies therein (which are by many believed to replication factories) and where vRNPs are formed and migrate to, in preparation for assembly, is the more exciting and relevant question. Do fast-moving vRNPs represent a major mechanism underlying assembly or artefacts? Experimentally, this may be the next dimension of study, however any discussion on how the data fits within the current understanding of assembly steps, and involvement of inclusion bodies, is lacking.

The question of assembly sites and budding sites is indeed not addressed in this work and is a subject for other studies. We have previously demonstrated that inclusion bodies (IBs) are the sites of viral RNA synthesis including the viral genome. Encapsidation is considered to take place during replication in IBs and it is accepted, but not experimentally demonstrated, that ribonucleocapsid association with P, L and M2-1 also takes place in IBs. We observe RNPs, that exit IBs which is in favor of this hypothesis. The following sentence has been added to the results.

Results Line 134-135 *Moreover, we observed some particles moving out of the IBs (arrowhead on Supplementary Movie 1)*

And Legends Supplementary movies line 94: *Arrowheads point a RNP moving out of an IB.*

The significance of rapid movement of RNPs on microtubules in the viral cycle is difficult to quantify. Depolymerization of MTs (remotely from the entry steps) induces a significant reduction in viral titer and we show that they are involved in this rapid transport suggesting that this transport is important for viral multiplication. However, it must be emphasized that 1) multiplication remains possible implying that other pathways exist and 2) that the cellular model is artificial. It is possible that in ciliated cells in which MTs are highly organized and key in transport to the apical pole, this transport mechanism is prevalent.

The following sentence has been added in the discussion

Discussion line 377-78: *Relative contribution of MT transportation into RSV assembly is yet to be determined.*

Several older papers, such as Kallewaard listed by the authors but also several other papers, have found that in addition to microtubules, cortical F-actin also plays a role in the late stages of assembly. These findings should be discussed in the paper.

These papers pointed the involvement of the Cortical F actin in RSV filaments assembly and budding. We believe the fast transportation to occur before this last stage. This hypothesis has been added in the discussion.

Discussion Lines 382-385: *Cortical F actin network has been involved in RSV filaments assembly and budding (23,33,42,43* e.g. Kallewaard *et al,* 2005; Jeffree *et al*, 2007 ; Ravi *et al,* 2013 ; Ravi *et al*, 2021*), this might suggest that RNPs are transported from the cytoplasmic IBs near the plasma membrane thanks to MT network before interacting with F actin partners to promote virion assembly and budding.*

References 42 and 43 have added

Although GFP-N is incorporated into vRNPs, we cannot know whether GFP affects N-coating of vRNPs or vRNP movement. For example, it cannot be excluded that GFP slows down vRNP movement, and that faster motion is under-represented. This does not diminish the findings but should be acknowledged.
We added the following sentence in the discussion

Line 345 *although we can not rule out that GFP-N incorporation into the RNPs impairs their movements*

Fig 7. In this paragraph, please indicate more clearly in the text which part of the figure is referred to.
Thanks for pointing out this ambiguity. This has been corrected by adding reference to the specific panels.

Lines 303 *Fig 7a*, 307*(Fig 7a, bound fraction)*, 310 *(Fig 7b)*, 315 *(Fig 7a; RSV-L-GFP).*

Fig. 7b. There seems to be only a very small amount of P, especially given the high amounts of HA-Rab11a and N on the same blot. This finding does not support that P is co-precipitated, contrary to the statement in line 307.

Thanks for pointing this out. This is an artefact due to the strong signal of the N antibody in this blot. We reloaded the sample of this experiment for a new blot (with more diluted N antibody) and modified the figure accordingly. Please find below the old figure, the new one (version 1) and a second version in which P and N were revealed separately.

## Part III – Minor Issues: Editorial and Data Presentation Modifications Please use this section for editorial suggestions as well as relatively minor modifications of existing data that would enhance clarity.

Reviewer #1: A few grammatical changes are suggested on the attached manuscript copy.

We are grateful to reviewer 1 for correcting our mistakes in English. All recommended corrections have been done.

Regarding the following question: 138-137 what are instant speeds and maximal instant speed?

We specify the definition of instant speed and of the other parameters in Fig2 legend

Legend Fig 2 line 172-176*:* instant speed *(ratio between the minimal distance between two positions and time interval between these positions),* track length *(sum of the minimum distance between 2 consecutives positions for the whole track),* track displacement *(minimal distance between the first and the last position of the particle).*

Reviewer #2:

- line 67: add reference: Reference 9 has been added
- lines 275 and 276: define ER. Do the authors mean RE? Yes we did mean RE, thanks for the correction.
- Fig 6: define the difference between * and **. Done: *** p*< 0.01, *** *p*<0.05 using t test with Welch’s correction. One star was missing.
- line 337-338. Recent work by Piedra et al suggests the transcription gradient (and protein levels) may be more complex than anticipated. Hence, it cannot be predicted whether N-GFP levels would be lower than N levels.

We are using the Long strain in which the transcriptional gradient still seems to exist even though less pronounced than for paramyxoviruses. Anyway, the blot (fig 7a) shows that GFP-N expression is lower than the wild type. We modified the sentence in the discussion to stress that transcriptional gradient is discussed for RSV.

Lines 341-42: *GFP-N is less abundant than the wild-type N (Fig 7a) which might result from the transcription gradient.*

- line 381: first use of ERC should be defined. Thanks. Replaced by RE.

**Additional changes:**

Bibliographic references 27 and 34 were referring to the same paper. This mistake has been corrected.
